# Dietary, physical activity, and weight management interventions among active-duty military personnel: a systematic review

**DOI:** 10.1186/s40779-018-0190-5

**Published:** 2018-12-24

**Authors:** Ahmad M. Malkawi, Ree M. Meertens, Stef P. J. Kremers, Ester F. C. Sleddens

**Affiliations:** 1 Department of Health Promotion, School of Nutrition and Translational Research in Metabolism (NUTRIM), Maastricht University Medical Center+, PO Box 616, Maastricht, 6200 MD the Netherlands; 2 Department of Health Promotion, School of Nutrition and Translational Research in Metabolism (NUTRIM), and Care and Public Health Research Institute (CAPHRI), Maastricht University Medical Center+, PO Box 616, Maastricht, 6200 MD the Netherlands

**Keywords:** Dietary, Physical activity, Weight loss, Interventions, Military, Systematic review

## Abstract

**Background:**

Research has been conducted to assess the effectiveness of weight management, dietary and physical activity interventions in military settings. However, a recent and comprehensive overview is lacking. The aim of this systematic review is to examine the evidence and describe key components of effective interventions in terms of improving body composition, dietary behaviors, and physical activity among active-duty military personnel.

**Methods:**

PubMed, PsycInfo, and CINAHL were searched on the 17th of November 2017 to identify interventions that promoted diet and/or physical activity among active-duty military personnel. Studies were included if they assessed outcomes related to anthropometric measurements, dietary behaviors, or fitness/physical activity levels. There were no restrictions regarding publication date, follow-up duration, and sex. After screening, a total of 136 studies were eligible. Of these studies, 38 included an educational and/or behavioral change component, and 98 had only physical or fitness training as part of basic military training. Only studies that included an educational and/or behavioral change component were assessed for quality using the Effective Public Health Practice Project tool and included in the qualitative synthesis of the results.

**Results:**

Based on consistent evidence from studies that were rated as moderate or strong, there is good evidence that military weight management interventions are effective in improving body composition for durations of up to 12 months. Effective interventions are more likely to be high intensity (have a greater number of sessions), are more often delivered by specialists, and use theoretical base/behavioral change techniques and a standardized guideline. Dietary interventions can potentially reduce total fat and saturated fat intake. Dietary interventions that target the kitchen staff and/or increase the availability of healthy food are more likely to be effective in the short term. The results regarding military physical fitness interventions were inconclusive.

**Conclusion:**

Despite limitations such as the diversity and heterogeneity of the included interventions, outcome measurements, and follow-up duration, this systematic review found good evidence that weight management interventions are effective, especially in terms of weight loss. More studies are needed to acquire solid evidence for effectiveness for durations longer than 12 months and to identify key components of the effective dietary and physical activity educational and/or behavioral change interventions, especially in countries outside Europe and the US.

**Electronic supplementary material:**

The online version of this article (10.1186/s40779-018-0190-5) contains supplementary material, which is available to authorized users.

## Background

Obesity is a significant public health problem worldwide. According to WHO statistics, the global prevalence of overweight for adults aged 18 years or older was 39% in 2014 [1]. It is estimated that 1.9 billion adults aged 18 years and over were overweight, and 600 million of them were obese in 2014 [2]. The number of obese and overweight adults is expected to reach 2.7 billion adults in 2025 [3]. This rising trend among civilians has adverse effects on the military, as demonstrated by hindering the recruitment and maintenance of military manpower [4]. Evidence from the US showed that the rising trend of obesity was also observed in the military population [5]. In 2012, the prevalence of overweight and obesity was 49.3 and 19.4%, respectively, among active-duty soldiers in the US [6]. According to data collected in 2005 in the US for active-duty soldiers, obesity was significantly higher among Navy and Airforce military personnel [4]. Physical fitness, weight, and body composition standards are very relevant to military occupations [5]. Maintaining these standards is important to ensure that military personnel are capable of handling the physical demand in the military environment and reducing the risks of injuries in addition to maintaining proper military appearance. Despite these standards, military personnel are still at risk of weight gain as they are exposed to unhealthy food (e.g., energy dense food), which is more accessible and convenient, especially during deployment and relocation. Moreover, the current military environment is not always conducive to regular exercise.

A review that assessed the determinants of obesity in the military population found that obesity is significantly higher among men, people over 35 years of age, individuals with lower ranks, and married officers, in addition to certain ethnic groups such as African-American and Hispanic ethnic groups [7]. Along with common consequences such as type 2 diabetes and hypertension, overweight and obesity are linked to reduced quality of life, reduced work productivity and joint and back disorders [8]. Moreover, obesity has a significant economic impact in the military setting. It is estimated that obesity costs the US military approximately 1.1 billion USD per year [9].

Obesity is a complex, multifactorial condition that develops from social, behavioral, metabolic, and physiological factors [10]. Therefore, a comprehensive approach for treating obesity can include strategies (e.g., nutrition education), programs, and environmental change (e.g., policies and changing the workplace food) [11]. Changing the environment can be associated with healthier eating behaviors [12], and interventions based on theories such as self-regulation have shown potential effectiveness [13]. The Veterans Affairs (VA) and Department of Defense (DoD) provided primary care clinicians with clinical guidelines for screening and treating overweight and obesity among military personnel [6]. The guidelines recommend using a comprehensive and interdisciplinary approach that combines dietary, physical activity and behavioral components. These interventions are mainly designed to help military personnel who fail to meet fitness and body weight standards [5].

A preliminary search revealed research examining the effectiveness of lifestyle and weight management interventions that aim to maintain weight and fitness standards among military populations [14–17]. Sanderson et al. [7] conducted a systematic review to assess the effectiveness of weight management interventions in the military setting. Sanderson et al.’s review was conducted in 2011 and only included 13 studies that aimed primarily to treat obesity in the military population. The scope of the current review is broader and includes all dietary and physical activity interventions in addition to weight management interventions. Dietary behaviors and fitness tests are included along with anthropometric measurements.

This review will focus on lifestyle interventions that contain a more structured and advanced effort for treating and preventing obesity than standard and regular physical training in the military. The aim of this review is to assess the effectiveness of dietary and physical activity interventions among active-duty military personnel in terms of improving dietary behaviors, fitness level, and body composition measures such as BMI and body fat percentage. It also aims to identify key components that are associated with improving weight and/or behavioral change, such as the delivery mode (such as group or individual counseling, self-help materials and internet-based advice), intensity (number of contacts), duration of the intervention, and theoretical underpinning.

## Methods

### Search strategy

This systematic review was conducted according to PRISMA guidelines (Preferred Reporting Items for Systematic Reviews and Meta-Analyses) [18]. An electronic search was conducted on 17 November 2017 using the PubMed, PsycInfo (via EBSCO), and CINAHL (via EBSCO) bibliographic databases. Specific controlled search terms were used for each database to identify potential studies. For instance, MeSH terms in PubMed and Thesaurus in PsycInfo and CINAHL were used to identify synonyms for each key concept. A combination of three key concepts was applied to the search strategy. The first key concept was related to interventions or programs, the second to outcome measurements, and the third to the military population. The search terms were reviewed by all authors. The search was limited to human studies published in English and Arabic. There was no restriction regarding the publication date. The same search strategy was applied to all 3 databases (see Appendix for the PubMed detailed search strategy).

Studies were included if they met the following criteria, which were established using the PICOS (participants, interventions, comparison, outcomes and study designs) strategy [18]:

### Participants

Studies were included if the target population was active-duty military personnel from any country. Studies were excluded if the target population was veterans, military family members, retired military people and military high school students. Participants in the included studies could have been in a variety of military settings to include basic military training or service members stationed at various installations and were in the Navy, Army, Marines, Air Force, and Coast Guard, but not the Reserve and National Guard forces because these individuals are civilians most of the time. We did not apply restrictions regarding the initial weight status. In addition, participants could have been of any sex and ethnic background.

### Interventions

Interventions were included if they provided any form of counseling or education or targeted the environment such as the canteens or restaurants inside military bases. Interventions could have promoted nutrition, physical activity, or both. Interventions that focused solely on providing dietary supplements were excluded.

### Comparison

Studies with or without a control group were included.

### Outcomes

Studies were included if they involved at least one of the following outcomes: anthropometric measurements (such as weight, BMI, body fat percentage, waist circumference), observable food and dietary behaviors (such as fruit and vegetables, carbohydrates, fibers, and sugar intake), and physical activity or fitness measurements (such as running time, total Metabolic equivalent of task (MET), and Maximal oxygen consumption (VO_2__max_)). Outcomes could have been measured subjectively (e.g., self-reported dietary questionnaire) or objectively by using weighing scales for anthropometric measurement or pedometer and accelerometer for assessing physical activity level. Outcomes such as self-efficacy, attitude, motivation, quality of life, nutrition knowledge, and nonobservable dietary intake (e.g., micronutrient intake) in addition to cardiovascular metabolic risk factors such as cholesterol level, blood pressure, and hormonal change measurements were excluded as they are not related to anthropometric measurements or actual behavior.

### Study design

There were no restrictions regarding the length of follow-up. Randomized control trials (RCTs) as well as non-RCTs and pretest-posttest studies were included. Posttest study designs, case studies, and qualitative studies were excluded because they do not provide enough evidence to assess effectiveness.

### Selection process

**Fig. 1:**
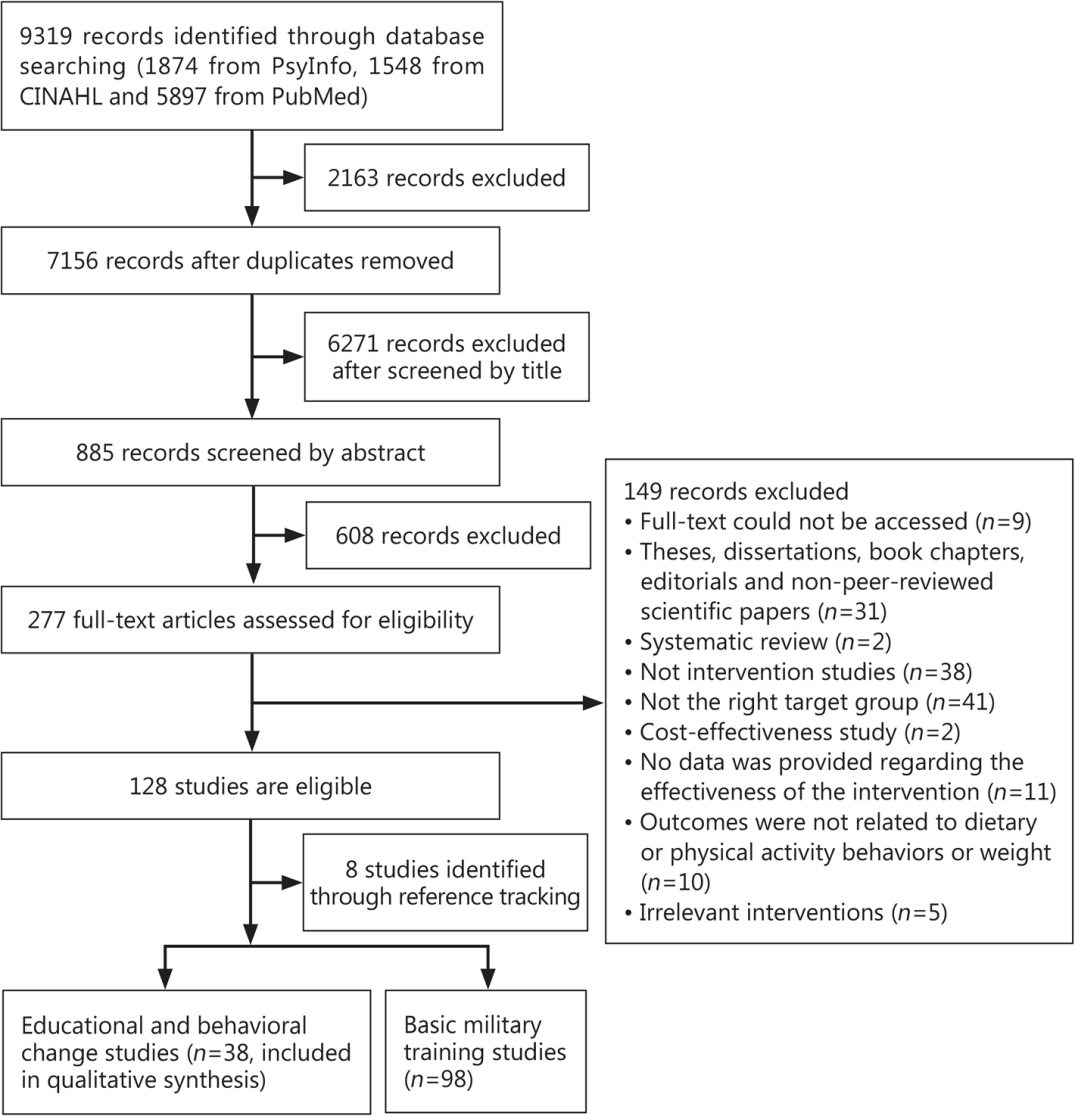
Flow diagram of literature search by database

Figure 1 summarizes the screening process for this systematic review. A total of 9319 relevant citations were identified from the three databases: 5897 articles from PubMed; 1874 articles from PsycInfo; and 1548 articles from CINAHL. After combining citations from all databases and removing duplicates (*n* = 2163), a total of 7156 articles were eligible for title screening. All subsequent screening of the citations was performed by two reviewers. One reviewer (AM) screened all titles and abstracts, while the second screening was performed by YS, ES or RM. The screening was based on the eligibility criteria. Any disagreement between reviewers during title and abstract screening was resolved by including the articles in the subsequent screening process. After screening 7156 titles, 885 citations were eligible for abstract screening. Following the screening of 885 abstracts, 277 articles were selected for full-text assessment. Full-text assessment was performed by two reviewers independently, and any disagreement during this stage was resolved by discussion between the four reviewers (AM, RM, ES, and YS). Studies that did not meet the inclusion criteria (*n* = 149) were excluded. Figure 1 displays the reasons for exclusion. Of these 149 articles, 9 could not be accessed even though we used more than one university library and contacted the corresponding author. After the full-text assessment, a total of 128 articles were eligible. The reference lists of the finally selected articles were screened to identify additional relevant articles. This resulted in the inclusion of another eight articles (*n* = 136).

### Data extraction

The final selection of 136 studies was divided into two types: (1) educational and behavioral change interventions and (2) basic military training interventions that did not include additional educational/behavioral interventions on diet and/or physical activity. Given this intervention heterogeneity and considering the focal point of this review was on interventions that focused on weight management and behavioral change, we decided to restrict the data extraction and quality assessment to the educational and behavioral change interventions and excluded studies that focused solely on the physical or fitness training activities that are part of basic military training. A full list of basic military training studies that were excluded is available in Additional file 1. A standardized data extraction form was developed by the authors of this review and pretested using a few eligible studies. Amendments were performed as suggested by all authors. Data were extracted by one reviewer. Extracted data from all finally selected studies included author name, study design, aim of the study, inclusion and exclusion criteria, baseline characteristics of the target population, recruitment process (randomization, blinding, unit of allocation), detailed description of the intervention (setting/country of the intervention, duration, intensity, number of contacts, delivery mode, theoretical basis, and intervention provider), type of control condition (standard care or placebo), outcome measurements (outcomes, definitions, measurement tool, validity and reliability of the tool, unit of measurement), timing of follow-up, attrition rate, the results, and subgroup analyses. To determine intervention effectiveness, a study first must have reported a significant change (*p* < 0.05) over the intervention period in body weight or (BMI) or any other anthropometric measures (such as body fat percentage), in dietary intake, including fruits and vegetables, total energy and total fat intake, or in fitness-related measures such as running time and VO_2_ max. Furthermore, effect sizes were calculated, if possible, for weight measurements. Moreover, changes in anthropometric data are reported to provide insight into the clinical relevance of these effects.

### Quality assessment

The quality of the educational and behavioral change intervention studies (*n* = 38) was assessed using the Effective Public Health Practice Project (EPHPP) tool [19]. The construct validity of this tool is described elsewhere [20]. The tool contains questions that evaluate six main components for each study: selection bias, study design, confounders, blinding, data collection methods, and withdrawals/drop outs. Each component was rated as weak, moderate, or strong according to a standardized guide and dictionary. For instance, a study was rated as strong for the study design component if it is an RCT or a controlled clinical trial. Cohort, case-control, or interrupted time series designs were rated as moderate, while other designs were rated as weak. The overall rating for each study was assessed as weak, moderate, or strong according to the rating of each component. The overall rating was strong if there were no weak ratings and at least four strong ratings. A moderate overall rating meant one weak rating and less than four strong ratings. A weak overall rating had two or more weak ratings. The quality assessment rating was conducted independently by two reviewers (AM and YS). Any inconsistency was resolved by discussion between them.

## Results

### Study characteristics

In total, 38 studies were selected and included in the qualitative synthesis. The majority of studies were conducted in the US [14, 17, 21–42], followed by Europe [43–54]. Ten studies included recruits during basic military training [21, 25, 43, 44, 47, 48, 50, 52, 55, 56], while 28 included fully qualified military personnel [14, 17, 22–24, 26–42, 45, 46, 49, 51, 53, 54]. Included studies were conducted in different military branches, including the Navy [14, 22, 26, 32], Air Force [17, 23, 30, 31, 34, 40–42], Army [33, 35–38, 49, 51, 53, 54], Defense Force [46], multiple military branches [24, 26–29] and military service/basic military training [21, 25, 43, 44, 47, 48, 50, 52, 55, 56]. Twelve studies were RCTs [14, 23, 25, 30, 32, 33, 35–37, 41, 51, 52], nine studies were nonrandomized control trials or quasi-experimental studies [17, 26, 31, 43, 44, 46, 47, 50, 52], 13 studies were longitudinal studies [21, 22, 24, 28, 38–40, 45, 48, 49, 54–56], and four studies were retrospective studies [27, 29, 34, 53]. All studies were published in English from 1975 onwards. The sample size of the participants ranged from 18 to 68,591, and ages were 18 years or older. Study duration ranged from six weeks to five years; 23 studies conducted follow-up measurements for six months or less [14, 23, 24, 26, 27, 30–36, 38, 40–42, 44, 46, 47, 50, 52, 55, 56], and 14 studies conducted follow-up for more than six months [17, 21, 22, 28, 29, 37, 39, 43, 45, 48, 49, 51, 53, 54]. The follow-up duration was not stated by one study [25] (see Additional file 2).

### Quality of the included studies

Quality assessment was conducted for 38 studies. A total of 28 studies received a weak rating [21, 23–28, 32–36, 42, 43, 45–50, 53–56], eight received a moderate rating [17, 22, 29, 31, 37, 41, 44, 51], and two received a strong rating [14, 30]. The main cause of a weak rating was selection bias as the method of recruitment was not reported or the target population was not representative [21, 25–27, 32, 33, 35, 48, 50]. Moreover, a high attrition rate (more than 40%) was common [24, 27, 32, 33, 35, 36, 43, 49, 50] (see Additional file 2 and Additional file 3).

### Description of the included interventions

Seven studies only promoted a healthy diet [37, 44, 45, 50–52, 56], and six only promoted physical activity [21, 43, 46–48, 54, 55], while 24 studies promoted both diet and physical training [14, 17, 22–36, 38–42, 49, 53]. The use of theoretical frameworks or behavioral change techniques was reported by 18 studies [14, 17, 22, 24, 26–32, 34, 38–41, 56]. One study used motivational interviewing [30], while another used mindfulness meditation [51]. Goal-setting and self-monitoring were commonly used by four interventions [24, 28, 29, 31]. Some interventions used different self-monitoring tools to record and track activity level, caloric intake, and food intake, such as accelerometers [35], calorimeters [31], and diaries [30, 32]. The delivery mode of the interventions varied between studies. Counseling was provided by different modes, including group educational sessions (traditional [43, 48] or slide-based formats [34, 42]), individual counseling [37], telephone calls [30], log-in to an interactive webpage [26] or online lessons [30], and email messages [17]. Many interventions implemented regular physical fitness sessions (sport, aerobic, circuit, strength training) accompanied by educational sessions [21, 46, 47]. Some dietary interventions targeted the kitchens by increasing the availability of healthy food and training kitchen staff [37, 44, 45, 50, 52], while one used controlled menus or calorie-controlled meal plans [33]. Two interventions provided medication in addition to counseling for weight loss [29, 36]. Different educational supportive methods were provided, such as self-directed booklets [17], handouts [31], brochures and posters [50, 56], and food models [23]. There was large variation in the number of intervention sessions provided, ranging from 8 to 15 [32, 40, 42, 47, 56], 24–36 [14, 23, 51, 54], 40–48 [24, 26, 27, 29, 39, 43] and 72–480 [21, 22] total sessions. Five weight management interventions conducted an introductory intensive program that included full day sessions that lasted from 1 to 3 weeks [24, 26, 27, 29, 39] (see Additional file 2).

### Effectiveness of interventions with an educational or behavioral component

The effectiveness of the interventions was assessed according to outcome measurements. Outcomes were categorized into three main types: anthropometric, dietary, and fitness/physical activity-related outcome measurements. A narrative summary of the results will address each outcome within each main category (e.g., weight loss/BMI for the anthropometric category) according to follow-up duration. Many variables were assessed in relation to the interventions’ effectiveness, including features of interventions (e.g., intensity, delivery mode, theoretical base), intervention setting (e.g., Navy, Air Force), and the characteristics of the target population (ethnic background and sex).

### Effectiveness of military interventions aiming to reduce weight and body fat

A total of 27 studies (two rated strong, six moderate and 19 weak) aimed to improve body weight/BMI, waist circumference, body fat percentage and lean body mass.

#### Weight/BMI

Strong/moderate*-*rated studies showed a significant reduction in weight at different time points, including the 5-week [51], 3-month [31], 6-month [14, 30, 41, 51], and 12-month follow-ups [17, 22, 29]. Only one intervention reported weight reduction after 5 weeks and 6 months, but not after 12 months [51]. The effect size of weight reduction calculated was small and ranged from − 0.17 to − 0.26 [13, 28, 51], except for one intervention arm, which stated a large effect size for the cumulative weight loss equal to 0.71 [51]. The evidence from weak-rated studies is consistent with that from moderate/strong-rated studies. For instance, the majority of weak-rated studies showed a significant reduction in weight/BMI [23, 26–28, 33, 36, 38–40, 49, 53] with one study showing a significant reduction after 24 months in comparison to baseline [49]. Only two weak-rated studies found a significant increase in weight/BMI [54, 56]. However, one of them reported an increase in lean body mass. A total of 10 studies reported significant weight loss ranging from 0.5*–*2.8 kg [17, 22, 23, 30, 32, 38, 41, 43, 51, 54], four studies 3–3.8 kg [33, 36, 49, 53], and five studies 4.3–8.6 kg [14, 24, 27, 28, 31]; while two studies showed significant weight gain ranging from 1.1–1.9 kg [55, 56] (see Additional file 2).

#### Body fat percentage and lean mass

There was an improvement in body fat percentage after 6 months [30, 41] and one year [22]. Lean body mass was significantly increased after 9 [54] and 12 months [22]. Three weak*-*rated studies found a significant reduction in body fat percentage [38, 49, 53]. Six studies reported a reduction in body fat percentage ranging from 0.4–1.7% [30, 32, 33, 36, 41, 42]*,* and three studies reported a reduction ranging from 3.8–7.8% [14, 22, 38]; two studies reported an increase in lean body mass (≈1.5 kg) [22, 38].

#### Waist circumference

One strong/moderate*-*rated study [30] and three weak*-*rated studies [32, 49, 53] found a significant reduction in waist circumference, while one study found no significant reduction in waist circumference. The reduction in waist circumference ranged from 2.1–3.8 cm [30, 32, 49, 53].

#### Features of effective weight management interventions

A total of six moderate/strong-rated [14, 17, 22, 29–31] and 12 weak-rated studies [24, 26–28, 32, 34, 38–43] used a theory or behavioral change technique(s). Of these interventions, the most common theory/model was the social cognitive theory and the trans-theoretical model [17, 24, 26, 28, 34, 56]. In addition, one intervention used motivational interviewing counseling calls plus behavioral modification strategies such as self-monitoring, goal-setting, relapse prevention, and stimulus control [30]. Another study found that using indirect calorimetry for self-monitoring of calorie intake has significantly higher weight loss than usual care (weight loss was − 4.3 ± 3.3 for the intervention vs. –1.8 ± 3.2 kg for the control groups; *p* ≤ 0.05) [31]. One intervention that showed significant weight reduction in the short term (at 5 weeks and 6 months only) used mindfulness with self-compassion meditation [51]. Many interventions reported using only behavioral modification strategies [14, 22, 32].

A total of six moderate/strong-rated [14, 17, 22, 29–31] and 16 weak-rated studies [23, 24, 26–28, 32–34, 36, 38–42, 49, 53] promoted both diet and physical activity. One intervention only promoted diet [51], while another two interventions only promoted physical activity [21, 55]. The duration of interventions ranged from five weeks to one year [14, 17, 22, 29–31, 51]. The majority of interventions were high intensity [17, 21–24, 26–30, 32, 39, 40, 42]. Most of these interventions were conducted in the US and provide weekly follow-up sessions/lectures that extend from 3 to 24 months. For instance, Hunter et al. [30] and Dennis et al. [14] delivered weekly sessions over 6 months, while Earles et al. [29] and Robbins et al. [17] delivered weekly messages/sessions over 12 months. Mantzios et al. [51] and Trent et al. [22] delivered 2–4 weekly sessions over 12 months. However, one low-intensity intervention that used a weekly email with a self-directed booklet also showed a significant, if minimal annual weight reduction (1.3 Ib) [17]. Regarding the mode of delivery, the interventions used group sessions/lectures [14, 22, 24, 27–29, 31, 39, 51], printed educational materials [14, 17, 36, 41, 51], online educational resources [17, 30], and telephone calls [30]. Interventions that were effective in reducing body weight used individualized (e.g., one to one counseling) [30, 31, 36, 49], generic (not tailored) [17, 51], or both generic and individualized [29, 41] counseling to achieve behavioral change. A total of 20 interventions were delivered by a specialist, such as a dietitian [23, 31–33, 36, 40], psychologist [26, 29], or fitness trainer [22, 28]. Many studies reported using a curriculum or standardized guidelines [14, 17, 22, 25, 27–29, 31, 34, 35, 39, 41, 51].

The majority of studies included both men and women in their sample [17, 21, 22, 28–31, 34, 37, 51]. Three strong/moderate-rated and three weak-rated studies conducted subgroup analyses and found that the weight loss was not significantly different between males and females [22, 26, 28–30, 38]. Three moderate/strong-rated studies that found a significant improvement in weight-related outcomes were conducted in the US and included participants from different ethnic groups [22, 29, 30]. For instance, the LE3AN Program targeted different ethnic groups, including African-American, Caucasian, Hispanic, and Asian-American [29]. Two studies indicated that weight loss was not significantly different between ethnic groups [27, 30]. Moreover, many strong/moderate-rated dietary and weight management interventions were conducted in different military settings, including Air Force [17, 23, 31, 41] and Navy [14, 22, 26, 27, 32], in addition to multiple military settings [28, 29]. Interventions targeted recruits during the basic military training [21, 44, 55] and those who were professional soldiers [14, 17, 22, 23, 29, 30, 36, 37, 41, 49, 51] (see Additional file 2).

### Effectiveness of military interventions aiming to achieve changes in dietary behaviors

A total of four strong/moderate-rated [30, 31, 37, 44] and eight weak*-*rated [23, 25, 31, 42, 45, 50, 52, 56] studies aimed to improve dietary behaviors. Dietary behaviors were measured by validated self-reported tools, including the Arizona Food Frequency Questionnaire (AFFQ) [31], food diaries [44], and meat and snack screeners [30]*,* except for one study that used a digital photography method [37].

#### Whole grain and dietary fiber intake

One study stated that the whole-grain intake was significantly improved after five months [44], while another study found no significant improvement in whole-grain intake after 6 and 12 months but reported a significant reduction in refined grain at the 6- and 12-month follow-up [37]. Regarding weak*-*rated studies, dietary fiber intake as demonstrated by the cereal index and whole-grain intake was reported as improved by two studies [50, 52], while one study showed no improvement [56].

#### Fruit and vegetable intake

Two moderate/strong-rated studies showed some improvement in fruit and vegetable consumption after 5–6 months [30, 44], while one study found no significant improvement after 6 and 12 months [37]. Three weak*-*rated studies showed a significant improvement in fruit and vegetable intake [45, 50, 56], while two studies found no improvement [25, 52].

#### Fat intake

Two studies showed a significant reduction in energy derived from fat and saturated fat after 5–6 months [37, 44]. However, Crombie et al. reported a relapse after 12 months for fat and saturated fat at the 12-month follow-up [37]. Regarding weak-rated studies, five [23, 25, 42, 52, 56] showed a significant reduction in fat intake, and one did not [36].

#### Features of effective dietary interventions

Two strong/moderate-rated and three weak-rated studies that found a significant improvement in fat intake [37, 44, 52], refined grain intake [37], whole grain, fruit, and vegetables [44, 45, 50] targeted military base kitchens and focused on increasing the availability of healthy foods such as fruit, vegetables, and whole grains [37, 44, 45, 50, 52]. Training workshops were conducted to educate staff members about nutritional topics and healthy cooking methods [37, 44, 50, 52]. Supportive educational materials such as posters, color-coded nutrition information cards, and handouts were used to deliver nutritional messages [31, 37, 50]. Group educational and one-to-one online lessons were also used [23, 30, 31]. Two strong/moderate-rated interventions provided training on behavior modification strategies using the self-monitoring technique [30, 31]. Hunter et al. [30] promoted self-monitoring using a weight-tracking chart and submitting online food diaries, while MacDoniel et al. [31] used indirect calorimetry for calorie tracking. Two weak-rated studies used social cognitive theory [42, 56] (see Additional file 2).

### Effectiveness of military interventions aiming to improve fitness and physical activity level

Three strong/moderate-rated studies evaluated fitness and physical activity-related outcomes [30, 31, 41]. Three interventions mainly promoted physical activity by self-monitoring strategies using exercise diaries [30] and calorimeters [31]. Their outcomes included the total MET minutes/week, which was mainly measured by the International Physical Activity Questionnaire (IPAQ) or 7-day physical activity recall and the Army Physical Activity Test (APFT) [30], which is calculated by the sum of push-up, 2-mile run, and sit-up scores in addition to energy expenditure related to physical activity (kcal/d) [31]. Other outcomes included the maximum heart rate and VO_2_ max. Hunter et al. [30] found that an internet-based exercise intervention made no significant difference in total IPAQ in comparison with a usual care control group at the 6-month follow-up (17.4 ± 5 vs 16.5 ± 4.7, respectively). MacDoniel et al. [31] found no significant change in energy expenditure related to physical activity at the 3-month follow-up among the weight control group in comparison with a usual care group. The weight control group received training in basic nutrition, self-monitoring of food intake and exercise, physical activity, and behavior modification. Veverka et al. found no significant improvement in VO_2_ score [41]. Regarding weak-rated studies, three studies found a significant improvement in VO_2_ max [21, 43, 46]. In addition, three weak-rated studies found a significant improvement in sit-ups and push-ups [24, 34, 43], while one study did not [54]. The 2-mile run time was improved in James et al. [24], but not in Hickey et al. [46]. The maximum heart rate was improved in Hickey et al. [46], but not in Shrestha et al. [35]. Regarding those interventions that found a significant improvement in some fitness outcomes, four of them were high intensity [21, 24, 34, 43], and two used the cognitive behavioral approach [24, 34] (see Additional file 2).

## Discussion

This systematic review aimed to update evidence on weight management and dietary and physical activity interventions and tried to identify key components of effective interventions. It included a total of 38 studies with an educational and/or behavioral change intervention. Of these studies, 28 were rated weak, eight moderate, and two strong based on the EPHPP tool. This systematic review found good evidence that weight management military interventions were successful in improving body composition, especially in reducing weight and body fat percentage over 12 months. Effectiveness did not vary according to military setting, ethnicity, or sex. Few strong/moderate-rated dietary interventions reported a significant reduction in fat and saturated fat intake in the short term (over 6 months). This review could not draw any conclusions regarding the effectiveness of educational/behavioral interventions aiming to improve fitness or physical activity level due to a lack of evidence from strong/moderate-rated studies.

Based on the findings of this systematic review, it is recommended that weight management military interventions be of high intensity. The US Preventive Services Task Force defined high-intensity interventions as those that include more than one session per month in the first 3 months [57]. We found indications that these interventions are more effective when they are delivered by specialists (such as a dietitian or fitness instructor), use a theoretical basis or theory-based behavior change techniques (e.g., self-monitoring and goal-setting), promote both diet and physical activity, and use a standardized guideline or curriculum. Different delivery modes can be used, such as printed material, online sources, group and individual counseling sessions. Note that a lack of comparative studies (e.g., theory-based vs not theory-based interventions) prevented us from drawing firm conclusions. Promising results were obtained with dietary interventions that used self-monitoring and targeted the kitchen in military bases by supplying healthy food and training kitchen staff members.

This review updated a systematic review conducted by Sanderson et al. [7] to assess the effectiveness of weight management interventions in a military setting. Sanderson and colleagues found that successful interventions used behavioral change strategies such as self-monitoring, combined dietary and physical activity therapy, and structured follow-up. Our review findings are consistent with those of Sanderson et al. [7]. However, we included a higher number of studies and identified other key components of effective interventions, especially related to dietary and weight management interventions. The new findings included using a standardized guideline or curriculum and guidance by specialists. These components are suggested due to the complexity of overweight and obesity and the need for comprehensive interventions that provide multiple behavioral management activities, address barriers to change and encourage prevention strategies [6]. Moreover, weight loss is improved with more sessions [57]. Regarding dietary interventions, targeting the kitchen staff and/or supplying healthy food was found to be the most important component.

Recommendations for implementing military weight management interventions are consistent with other settings [13]. One systematic umbrella review found that greater effectiveness of dietary/physical activity interventions was associated with engaging social support, targeting both diet and physical activity, increasing contact frequency, and using self-regulatory behavior change techniques (e.g., goal-setting, self-monitoring). Effectiveness was not associated with intervention setting, delivery mode, delivery provider, age, or sex. This is largely in line with the conclusions we could draw from intervention studies in the military setting. There were a few exceptions. For instance, one systematic review found no association with delivery provider and effectiveness in the primary care setting. It also found that certain ethnicities, such as white Caucasian, can achieve greater weight loss [13]. Additionally, our review could not find enough evidence to support using medications or meal replacement, which showed effectiveness in other settings [58, 59].

To enhance the effectiveness of dietary interventions, Taylor et al. conducted a review of dietary interventions in different settings (e.g., work and community setting) and recommended considering the barriers that prevent dietary modification [60]. In addition, interventions should use clear, concise, and achievable nutritional messages. Messages should be based on the participants’ need and presented in a fun and engaging manner.

Further research is needed to assess the long-term (more than one-year follow-up) effectiveness of weight management interventions. One of the key findings of our review is that studies in a military setting rarely have a follow-up period of longer than 12 months. This may partly be because military recruits move to other military branches and are more difficult to follow-up after that. However, studies among professional military usually do not have follow-ups after a year. A period of 12 months is too short for studying the sustainability of the intervention effects. For conscripts, the transfer to civil society may contribute to weight regain. Evidence from other settings showed that approximately 35% of participants gained weight after the first year of treatment [61]. Comparative research studies can be conducted to confirm the effectiveness of theory-based interventions (theory vs. not theory-based), high-intensity intervention (high intensity vs. low intensity), and those delivered by a specialist (specialist vs. nonspecialist). Further research could confirm the minimum thresholds of contacts/follow-ups needed to provide adequate advice and prevent relapse. There is a need for evidence from countries other than the US and Europe and to include other ethnic backgrounds.

Many studies relied on self-reported tools, especially for assessing physical activity and dietary behaviors. Many studies used subjective methods such as self-reported questionnaires, which may lead to self-reported bias [62]. It is recommended that physical fitness military interventions use standardized, valid, reliable, and common military fitness tests that cover all fitness components to facilitate comparison between studies [63, 64]. Such tests can include the Army Physical Fitness Test, which measures aerobic capacity and muscular strength/endurance. It consists of a two-mile run, push-ups, and sit-ups [65]. Many researchers consider VO_2_ max the best objective measure of cardiorespiratory fitness. However, VO_2_ max measurements require expensive equipment and trained technicians [62]. Other objective methods include heart rate monitors, pedometers, and accelerometers [63]. Dietary behaviors were mainly assessed by subjective methods such as dietary recall, food frequency assessment, and food diary or log. All these methods are subject to recall and estimation bias. Moreover, no single method is perfect for assessing dietary behaviors. It is recommended to use valid, reliable, culturally adapted, and efficient tools [66]. Dietary assessment methods should be selected in accordance with the intervention objectives and available resources [67].

The quality of studies can be improved by recruiting a representative sample of the target population to reduce selection bias and by including a control group, although this is challenging in the military setting [20, 68]. It is also recommended to conduct an intention-to-treat analysis to adjust for attrition and to report the effect size rather than only the *p*-value [69].

### Limitations

This systematic review has some limitations. A few potentially relevant articles could not be accessed despite trying to search more than one university’s library and contacting the authors. Moreover, data extraction was performed by one reviewer. There is also a methodological issue regarding using the EPHPP tool for quality assessment. For instance, it was difficult to assess the rating of some components such as selection bias and the representative sample for several studies due to limited information from studies. Some issues are related to the included studies and potentially affected our conclusions. Intervention fidelity/integrity was not reported by many studies. Many studies did not provide enough description about the interventions, making it difficult to draw a conclusion. The studies included were diverse and heterogeneous in terms of the type of intervention, outcome measurements, and follow-up duration, which made it difficult to assess effectiveness, especially for dietary and physical fitness interventions. Finally, most of the included studies were conducted in the US and Europe, which may limit the generalizability of our findings.

## Conclusion and recommendations

This systematic review found that weight management interventions are effective in improving body weight standards among active duty military personnel. It also identified potential key components that can improve the effectiveness in terms of weight loss. The evidence showed that such interventions in a military setting would save costs and reduce the direct medical cost [70, 71]. The review found a promising effectiveness of dietary interventions for certain outcomes such as fat intake in the short term. However, there is a need for more rigorous research to (1) evaluate the effectiveness of weight management interventions in the long term (after one year), (2) identify and confirm key components of dietary interventions that improve a wide range of dietary habits (e.g., sugar intake and meal pattern) in the long term, and (3) identify key components of military fitness interventions. There is a need for evidence from countries other than the US and Europe.

### Additional files

Additional file 1: This is a full list of basic military training studies. (DOCX 81 kb)

Additional file 2: Full description of included interventions, outcome measurements, results in addition to sample size, age, setting and quality rate. (DOCX 100 kb)

Additional file 3: Quality Assessment results for all included studies. (DOCX 22 kb)

## Appendix

**Table 1:** PubMed search strategy, 17 November 2017

Set	Search terms
#1	((“Program Evaluation”[Mesh]) OR (“Health Education”[Mesh]) OR (“health education”[Tiab]) OR (“Health Promotion”[Mesh]) OR (“health promotion” [Tiab]) OR (intervention[Tiab]) OR (interventions[Tiab]) OR (interventional[Tiab]) OR (program[Tiab]) OR (programs[Tiab]) OR (programme[Tiab]) OR (programmes[Tiab]) OR (effect[Tiab]) OR (effects[Tiab]) OR (effective[Tiab]) OR (efficacy[Tiab]) OR (impact[Tiab]) OR (impacts[Tiab]) OR (impacted[Tiab]) OR (impacting[Tiab]) OR (evaluate[Tiab]) OR (evaluated[Tiab]) OR (evaluates[Tiab]) OR (evaluation[Tiab]) OR (evaluating[Tiab]) OR (assess[Tiab]) OR (assessed[Tiab]) OR (assesses[Tiab]) OR (assessing[Tiab]) OR (assessment[Tiab]) OR (influence[Tiab]) OR (influenced[Tiab]) OR (influences[Tiab]) OR (influencing[Tiab]) OR (prevent[Tiab]) OR (prevented[Tiab]) OR (prevents[Tiab]) OR (preventive[Tiab]) OR (prevention[Tiab]) OR (preventing[Tiab]) OR (“Physical Education and Training”[Mesh]) OR (“physical education”[Tiab]) OR (“Exercise Therapy”[Mesh]) OR (“Weight Reduction Programs”[Mesh]) OR (“weight reduction”[Tiab]) OR (“obesity treatment”[Tiab]) OR (“weight management”[Tiab]) OR (“weight control” [Tiab]) OR (“weight maintenance”[Tiab]) OR (“Nutrition Therapy”[Mesh]) OR (“Diet Therapy”[Mesh]) OR (counsel[Tiab]) OR (counsels[Tiab]) OR (counseling[Tiab]) OR (therapy[Tiab]) OR (control[Tiab]) OR (regulation[Tiab]) OR (regulations[Tiab]) OR (regulate[Tiab]) OR(regulating[Tiab]) OR (management[Tiab]) OR (guidance[Tiab]) OR (recommendation[Tiab]) OR (recommendations[Tiab]) OR (strategy[Tiab]) OR (strategies[Tiab]) OR (strategic[Tiab]))
#2	(“Food Habits”[Mesh]) OR (“food habit”[Tiab]) OR (“food habits”[Tiab]) OR (“food intake”[Tiab]) OR (vegetable[Tiab]) OR (fruit[Tiab]) OR (vegetables[Tiab]) OR (fruits[Tiab]) OR (“Life Style”[Mesh]) OR (lifestyle[Tiab]) OR (diet[Tiab]) OR (diets[Tiab]) OR (dietary[Tiab]) OR (nutrition[Tiab]) OR (nutritional[Tiab]) OR (calorie[Tiab]) OR (calories[Tiab]) OR (caloric[Tiab]) OR (beverage[Tiab]) OR (beverages[Tiab]) OR (Drink[Tiab]) OR (Drinks[Tiab]) OR (Drinking[Tiab]) OR (Weight[Tiab]) OR (“Junk food”[Tiab]) OR (“Junk foods”[Tiab]) OR (“Fast food”[Tiab]) OR (“Fast foods”[Tiab]) OR (“Takeaway food”[Tiab]) OR (“Takeaway foods”[Tiab]) OR (“Processed food”[Tiab]) OR (“Processed foods”[Tiab]) OR (Fat[Tiab]) OR (Fatty[Tiab]) OR (“Energy dense”[Tiab]) OR (Sugar[Tiab]) OR (Sweet[Tiab]) OR (Sweets[Tiab]) OR (Sweetened[Tiab]) OR (Fiber[Tiab]) OR (Cereal[Tiab]) OR (“Whole meal”[Tiab]) OR (“Whole grain”[Tiab]) OR (Snack[Tiab]) OR (Snaking[Tiab]) OR (snacks[Tiab]) OR (meal[Tiab]) OR (meals[Tiab]) OR (portion[Tiab]) OR (portions[Tiab]) OR (serving[Tiab]) OR (servings[Tiab]) OR (weight[Tiab]) OR (“Body Weights and Measures”[Mesh]) OR (BMI[Tiab]) OR (obese[Tiab]) OR (obesity[Tiab]) OR (overweight[Tiab]) OR (“physical activity”[Tiab]) OR (exercise[Tiab]) OR (“Exercise”[Mesh]) OR (“Sports”[Mesh]) OR sport[Tiab] OR (sports[Tiab]) OR (“Sedentary Lifestyle”[Mesh]) OR (sedentary[Tiab]) OR (“Food and Beverages”[Mesh]) OR (fitness[Tiab]) OR (physical readiness[Tiab]) OR (intake[Tiab]) OR (“Diet”[Mesh]) OR (“Eating”[Mesh]) OR (“Overweight”[Mesh]) OR (“Body Mass Index”[Mesh]) OR (inactive[Tiab]) OR (inactivity[Tiab]) OR (“television view”[Tiab]) OR (“television viewing”[Tiab]) OR (“screen time”[Tiab]) OR (“screen use”[Tiab]) OR (“screen view”[Tiab]) OR (“screen viewing”[Tiab]) OR (“sitting time”[Tiab]) OR (“computer time”[Tiab]) OR (“computer use”[Tiab]) OR (“TV time”[Tiab]) OR (“TV use”[Tiab]) OR (“TV watch”[Tiab]) OR (“TV watching”[Tiab]) OR (“television watch”[Tiab]) OR (“television watching”[Tiab]) OR (“television use”[Tiab]) OR (“television time”[Tiab]) OR (“screen media”[Tiab])
#3	(Military[Tiab]) OR (“Armed Forces”[Tiab]) OR (Submariner[Tiab]) OR (Submariners[Tiab]) OR (army[Tiab]) OR (Navy[Tiab]) OR (Sailors[Tiab]) OR (Sailor[Tiab]) OR (“Coast Guard”[Tiab]) OR (marines[Tiab]) OR (“Airforce”[Tiab]) OR (recruits[Tiab]) OR (soldier[Tiab]) OR (soldiers[Tiab]) OR (“active duty”[Tiab]) OR (cadets[Tiab]) OR (“Military Personnel”[Mesh]) OR (“national service”[Tiab])
#4	Search (#1 AND #2 AND #3) Filters: Humans; English

## Abbreviations

AFFQ: Arizona Food Frequency Questionnaire
APFT: Army Physical Activity Test
EPHPP: Effective Public Health Practice Project
IPAQ: International Physical Activity Questionnaire
MET: Metabolic equivalent of task
VO_2 max_: Maximal oxygen consumption
