# Supplementary material for: Dietary, physical activity, and weight management interventions among active-duty military personnel: a systematic review
Source: Mil Med Res. 2018 Dec 24;5:43. doi: 10.1186/s40779-018-0190-5 (PMC6309065; doi:10.1186/s40779-018-0190-5)
Supplement: Supplementary file 1 — This is a full list of basic military training studies. (DOCX 81 kb) [file 40779_2018_190_MOESM1_ESM.docx]

Additional file 1:

This is a full list of basic military training studies [72-169]:

1. Organization WH. Noncommunicable diseases country profiles. 2014. 2014.

2. Riley L, Cowan M. Noncommunicable diseases country profiles. 2014. Geneva: World Health Organization. 2014.

3. Swinburn B, Dietz W, Kleinert S. A Lancet Commission on obesity. Lancet. 2015; 386(10005):1716-7.

4. Smith TJ, Marriott BP, Dotson L, Bathalon GP, Funderburk L, White A, et al. Overweight and obesity in military personnel: sociodemographic predictors. Obesity. 2012; 20(7):1534-8.

5. Tanofsky-Kraff M, Sbrocco T, Theim KR, Cohen LA, Mackey ER, Stice E, et al. Obesity and the US military family. Obesity (Silver Spring). 2013;21(11):2205-20.

6. Affairs DoV. VA/DoD clinical practice guideline for screening and management of overweight and obesity. In.: Office of Quality and Performance publication, Washington, DC; 2014.

7. Sanderson PW, Clemes SA, Biddle SJ. The correlates and treatment of obesity in military populations: a systematic review. Obes Facts. 2011; 4(3):229-37.

8. Gates DM, Succop P, Brehm BJ, Gillespie GL, Sommers BD. Obesity and presenteeism: the impact of body mass index on workplace productivity. J Occup Environ Med. 2008; 50(1):39-45.

9. Dall TM, Zhang Y, Chen YJ, Wagner RC, Hogan PF, Fagan NK, et al. Cost associated with being overweight and with obesity, high alcohol consumption, and tobacco use within the military health system's TRICARE prime-enrolled population. Am J Health Promot. 2007, 22(2):120-39.

10. [No authors listed]. Clinical guidelines on the identification, evaluation, and treatment of overweight and obesity in adults: executive summary. Expert Panel on the Identification, Evaluation, and Treatment of Overweight in Adults. Am J Clin Nutr. 1998, 68(4):899-917.

11. Lobstein T, Swinburn B. Health promotion to prevent obesity. In: Global perspectives on health promotion effectiveness. Springer New York, New York, NY, 2007; 125-150.

12. Eldridge JD, Devine CM, Wethington E, Aceves L, Phillips-Caesar E, Wansink B, et al. Environmental influences on small eating behavior change to promote weight loss among Black and Hispanic populations. Appetite 2016, 96:129-37.

13. Greaves CJ, Sheppard KE, Abraham C, Hardeman W, Roden M, Evans PH, et al. Systematic review of reviews of intervention components associated with increased effectiveness in dietary and physical activity interventions. BMC Public Health. 2011; 11:119.

14. Dennis KE, Pane KW, Adams BK, Qi BB. The impact of a shipboard weight control program. Obes Res. 1999; 7(1):60-7.

15. Veverka DV, Anderson J, Auld GW, Coulter GR. Use of the stages of change model in improving nutrition and exercise habits in enlisted Air Force men. Mil Med. 2003; 168(5):373.

16. Bathalon GP, McGraw SM, Sharp MA, Williamson DA, Young AJ, Friedl KE. The effect of proposed improvements to the Army Weight Control Program on female soldiers. Mil Med. 2006; 171(8):800-5.

17. Robbins AS, Chao SY, Baumgartner N, Runyan CN, Oordt MS, Fonseca VP. A low-intensity intervention to prevent annual weight gain in active duty Air Force members. Mil Med. 2006; 171(6):556-61.

18. Liberati A, Altman DG, Tetzlaff J, Mulrow C, Gøtzsche PC, Ioannidis JP, et al. The PRISMA statement for reporting systematic reviews and meta-analyses of studies that evaluate health care interventions: explanation and elaboration. PLoS Med. 2009; 6(7):e1000100.

19. Effective Public Health Practice Project. (1998). Quality Assessment Tool For Quantitative Studies. Hamilton, ON: Effective Public Health Practice Project. [<https://merst.ca/ephpp/>].

20. Thomas BH, Ciliska D, Dobbins M, Micucci S. A process for systematically reviewing the literature: providing the research evidence for public health nursing interventions. Worldviews Evid Based Nurs. 2004; 1(3):176-84.

21. Daniels WL, Wright JE, Sharp DS, Kowal DM, Mello RP, Stauffer RS. The effect of two years training on aerobic power and muscle strength of male and female cadets. Aviat Space Environ Med. 1982 ;53(2):117-21.

22. Trent LK, Stevens LT. Evaluation of the Navy's obesity treatment program. Mil Med. 1995;160(7):326-30.

23. Gambera PJ, Schneeman BO, Davis PA. Use of the Food Guide Pyramid and US Dietary Guidelines to improve dietary intake and reduce cardiovascular risk in active-duty Air Force members. J Am Diet Assoc. 1995;95(11):1268-73.

24. James LC, Folen RA, Garland FN, Davis MK. A new frontier for clinical health psychologists: Our leadership role in the development and management of an inpatient weight management program. Prof Psychol: Res Pract. 1997; 28(2):146-52.

25. Fiedler ER, Cortner DM, Ktenidis H, Balch M. Healthy eating in practice: The US Air Force demonstration at basic military training. Appl Prev Psychol. 1999; 8(3):155-63.

26. James LC, Folen RA, Earles J. Behavioral telehealth applications in the treatment of obese soldiers: A feasibility project and a report on preliminary findings. Mil Psychol. 2001; 13(3):177-86.

27. Simpson M, Earles J, Folen R, Trammel R, James L. The Tripler Army Medical Center's LE3AN Program: a six-month retrospective analysis of program effectiveness for African-American and European-American females. J Natl Med Assoc. 2004; 96(10):1332-6.

28. Bowles SV, Picano J, Epperly T, Myer S. The LIFE program: a wellness approach to weight loss. Mil Med. 2006; 171(11):1089-94.

29. Earles JE, Kerr B, James LC, Folen RA. Clinical effectiveness of the LE3AN program: a military healthy lifestyle program. J Clin Psychol Med Sett. 2007, 14(1):51-7.

30. Hunter CM, Peterson AL, Alvarez LM, Poston WC, Brundige AR, Haddock CK, et al. Weight management using the internet: a randomized controlled trial. Am J Prev Med. 2008; 34(2):119-26.

31. McDoniel SO, Nelson HA, Thomson CA. Employing RMR technology in a 90-day weight control program. Obesity Facts. 2008; 1(6):298-304.

32. Shay LE, Seibert D, Watts D, Sbrocco T, Pagliara C. Adherence and weight loss outcomes associated with food-exercise diary preference in a military weight management program. Eat Behav. 2009; 10(4):220-7.

33. Smith TJ, Sigrist LD, Bathalon GP, McGraw S, Karl JP, Young AJ. Efficacy of a meal-replacement program for promoting blood lipid changes and weight and body fat loss in US Army soldiers. J Am Diet Assoc. 2010;110(2):268-73.

34. Webber BJ, Nelson MS, Gildengorin V. Indicators of sequential fitness assessment failures for Travis Air Force Base airmen who attend the Be Well Course. Mil Med. 2012, 177(3):302-7.

35. Shrestha M, Combest T, Fonda SJ, Alfonso A, Guerrero A. Effect of an accelerometer on body weight and fitness in overweight and obese active duty soldiers. Mil Med. 2013; 178(1):82-7.

36. Smith TJ, Crombie A, Sanders LF, Sigrist LD, Bathalon GP, McGraw S, et al. Efficacy of orlistat 60 mg on weight loss and body fat mass in US Army soldiers. J Acad Nutr Diet. 2012; 112(4):533-40.

37. Crombie AP, Funderburk LK, Smith TJ, McGraw SM, Walker LA, Champagne CM, et al. Effects of modified foodservice practices in military dining facilities on ad libitum nutritional intake of US Army soldiers. J Acad Nutr Diet. 2013; 113(7):920-7.

38. Davis MK. A comprehensive weight-loss program for soldiers. Mil Med. 1996, 161(2):84-8.

39. James LC, Folen RA, Page H, Noce M, Brown J, Britton C. The Tripler LE3AN Program: a two-year follow-up report. Mil Med. 1999; 164(6):389-95.

40. Reppart JT, Shaw CG. A conceptual and statistical evaluation of a new obesity treatment program in a military population. Mil Med. 1978; 143(9):619-23.

41. Veverka DV, Anderson J, Auld GW, Coulter GR, Kennedy C, Chapman PL. Use of the stages of change model in improving nutrition and exercise habits in enlisted Air Force men. Mil Med. 2003; 168(5):373-9.

42. Buffington BC, Melnyk BM, Morales S, Lords A, Zupan MR. Effects of an energy balance educational intervention and the COPE cognitive behavioral therapy intervention for Division I U.S. Air Force Academy female athletes. J Am Assoc Nurse Pract. 2016; 28(4):181-7.

43. Dyrstad SM, Soltvedt R, Hallén J. Physical fitness and physical training during Norwegian military service. Mil Med. 2006; 171(8):736-41.

44. Stea TH, Uglem S, Wandel M, Mansoor MA, Frølich W. Association between folate intake from different food sources in Norway and homocysteine status in a dietary intervention among young male adults. Br J Nutr. 2009; 102(06):899-906.

45. Thorsen AV, Lassen AD, Tetens I, Hels O, Mikkelsen BE. Long-term sustainability of a worksite canteen intervention of serving more fruit and vegetables. Public Health Nutr. 2010; 13(10):1647-52.

46. Hickey J, Donne B, O’Brien D. Effects of an eight week military training program on aerobic indices and psychomotor function. J R Army Med Corps. 2012; 158(1):41-6.

47. Hofstetter MC, Mäder U, Wyss T. Effects of a 7-week outdoor circuit training program on Swiss Army recruits. J Strength Cond Res. 2012; 26(12):3418-25.

48. Marić L, Krsmanović B, Mraović T, Gogić A, Sente J, Smajić M. The effectiveness of physical education of the Military Academy cadets during a 4-year study. Vojnosanit Pregl. 2013; 70(1):16-20.

49. Sammito S. Obesity intervention during a work health promotion: the Obesity Intervention Program of the German military forces. J Occup Environ Med. 2013; 55(7):728-31.

50. Uglem S, Råberg Kjøllesdal MK, Frølich W, Wandel M. Effect of a Nutrition Intervention on Intake of Vegetables, Fruits, and Semi Whole Grain Bread Among Low and High Consumers in the Norwegian National Guard. Mil Med. 2014; 179(9):1013-20.

51. Mantzios M, Wilson JC. Exploring mindfulness and mindfulness with self-compassion-centered interventions to assist weight loss: theoretical considerations and preliminary results of a randomized pilot study. Mindfulness. 2015; 6(4):824-35.

52. Bingham CM, Lahti-Koski M, Puukka P, Kinnunen M, Jallinoja P, Absetz P. Effects of a healthy food supply intervention in a military setting: positive changes in cereal, fat and sugar containing foods. Int J Behav Nutr Phys Act. 2012; 9:91.

53. Sammito S. Results of a course based obesity intervention program during work. Work. 2016; 53(3):661-7.

54. Tomczak A, Bertrandt J, Klos A, Klos K. Influence of military training and standardized nutrition in military unit on soldiers' nutritional status and physical fitness. J Strength Cond Res. 2016;, 30(10):2774-80.

55. Glick Z, Kaufmann NA. Weight and skinfold thickness changes during a physical training course. Med Sci Sports. 1975; 8(2):109-12.

56. Herzman-Harari S, Constantini N, Mann G, Lencovsky Z, Stark AH. Nutrition knowledge, attitudes, and behaviors of Israeli female combat recruits participating in a nutrition education program. Mil Med. 2013, 178(5):517-22.

57. Force USPST. Screening for and management of obesity in adults: US Preventive Services Task Force recommendation statement. In.; 2014.

58. Moyer VA, U.S. Preventive Services Task Force. Screening for and management of obesity in adults: US Preventive Services Task Force recommendation statement. Ann Intern Med. 2012; 157(5):373-8.

59. Noakes M, Foster PR, Keogh JB, Clifton PM. Meal replacements are as effective as structured weight-loss diets for treating obesity in adults with features of metabolic syndrome. J Nutr. 2004; 134(8):1894-9.

60. Taylor PJ, Kolt GS, Vandelanotte C, Caperchione CM, Mummery WK, George ES, et al. A review of the nature and effectiveness of nutrition interventions in adult males–a guide for intervention strategies. Int J Behav Nutr Phys Act. 2013; 10:13.

61. Wing RR, Phelan S. Long-term weight loss maintenance. Am J Clin Nutr. 2005; 82(1 Suppl):222S-5S.

62. Haskell WL, Kiernan M. Methodologic issues in measuring physical activity and physical fitness when evaluating the role of dietary supplements for physically active people. Am J Clin Nutr. 2000, 72(2 Suppl):541S-50S.

63. Caspersen CJ, Powell KE, Christenson GM. Physical activity, exercise, and physical fitness: definitions and distinctions for health-related research. Public Health Rep. 1985; 100(2):126-31.

64. Medicine ACoS. The recommended quantity and quality of exercise for developing and maintaining fitness in healthy adults (position statement). Med Sci Sports. 1978; 10(3): vii-x.

65. Knapik J. The Army Physical Fitness Test (APFT): a review of the literature. Mil Med. 1989; 154(6):326-9.

66. Miller MP. Best questions and tools for quickly assessing your patient's dietary health: Towards evidence-based determination of nutritional counseling need in the general medical interview. Nutr Noteworthy. 2005, 7(1).

67. Shim JS, Oh K, Kim HC. Dietary assessment methods in epidemiologic studies. Epidemiol Health. 2014; 36:e2014009.

68. Higgins JP, Green S. Cochrane handbook for systematic reviews of interventions, vol. 5: Wiley Online Library; 2008.

69. Sullivan GM, Feinn R. Using effect size-or why the P value is not enough. J Grad Med Educ. 2012; 4(3):279-82.

70. Hersey JC, Khavjou O, Strange LB, Atkinson RL, Blair SN, Campbell S, et al. The efficacy and cost-effectiveness of a community weight management intervention: a randomized controlled trial of the health weight management demonstration. Prev Med. 2012; 54(1):42-9.

71. Rasu RS, Hunter CM, Peterson AL, Maruska HM, Foreyt JP. Economic evaluation of an Internet-based weight management program. Am J Manag Care. 2010; 16(4):e98-104.

72. Vantarakis A, Chatzinikolaou A, Avloniti A, Vezos N, Douroudos II, Draganidis D, et al. A 2-month linear periodized resistance exercise training improved musculoskeletal fitness and specific conditioning of navy cadets. J Strength Cond Res. 2017;31(5):1362-70.

73. Ray U, Sinha B, Tomer O, Pathak A. Aerobic capacity & perceived exertion after practice of Hatha yogic exercises. Indian J Med Res. 2001; 114:215-21.

74. Patton JF, Daniels WL, Vogel JA. Aerobic power and body fat of men and women during army basic training. Aviat Space Environ Med. 1980; 51(5):492-6.

75. Marcinik EJ, Hodgdon JA, Mittleman K, O'brien JJ. Aerobic/calisthenic and aerobic/circuit weight training programs for Navy men: a comparative study. Med Sci Sports. 1985; 17(4):482-7.

76. Wilmore JH. Alterations in strength, body composition and anthropometric measurements consequent to a 10-week weight training program. Med Sci Sports. 1974, 6(2):133-8.

77. Lutz LJ, Gaffney-Stomberg E, Scisco JL, Cable SJ, Karl JP, Young AJ, et al. Assessment of dietary intake using the healthy eating index during military training. US Army Med Dep J. 2013; 91-7.

78. Woodruff SI, Conway TL, Linenger JM. An assessment of pre-and post-fitness measures in two remedial conditioning programs. Mil Med. 1992; 157(1):25-30.

79. Abt JP, Oliver JM, Nagai T, Sell TC, Lovalekar MT, Beals K, et al. Block-periodized training improves physiological and tactically relevant performance in naval special warfare operators. J Strength Cond Res. 2016; 30(1):39-52.

80. Pasiakos SM, Karl JP, Lutz LJ, Murphy NE, Margolis LM, Rood JC, et al. Cardiometabolic risk in US Army recruits and the effects of basic combat training. PLoS One. 2012; 7(2):e31222.

81. Faff J, Korneta K. Changes in aerobic and anaerobic fitness in the Polish army paratroopers during their military service. Aviat Space Environ Med. 2000; 71(9):920-4.

82. Vogel J, Crowdy J, Amor A, Worsley D. Changes in aerobic fitness and body fat during army recruit training. Eur J Appl Physiol Occup Physiol. 1978; 40(1):37-43.

83. Santtila M, Keijo H, Laura K, Heikki K. Changes in cardiovascular performance during an 8-week military basic training period combined with added endurance or strength training. Mil Med. 2008; 173(12):1173-9.

84. Marcinik E, Hodgdon J, Englund C, O'Brien J. Changes in fitness and shipboard task performance following circuit weight training programs featuring continuous or interval running. Eur J Appl Physiol Occup Physiol. 1987; 56(2):132-7.

85. Smoak B, Norton J, Ferguson E, Deuster P. Changes in lipoprotein profiles during intense military training. J Am Coll Nutr. 1990; 9(6):567-72.

86. Santtila M, Kyröläinen H, Häkkinen K. Changes in maximal and explosive strength, electromyography, and muscle thickness of lower and upper extremities induced by combined strength and endurance training in soldiers. J Strength Cond Res. 2009; 23(4):1300-8.

87. Christensen PA, Jacobsen O, Thorlund JB, Madsen T, Møller C, Jensen C, et al. Changes in maximum muscle strength and rapid muscle force characteristics after long-term special support and reconnaissance missions: a preliminary report. Mil Med. 2008; 173(9):889-94.

88. Faff J, Satora P, Stasiak K. Changes in the aerobic and anaerobic capacities of army recruits during their military training are related to the initial level of physical fitness of the subjects. Biol Sport. 2002; 19(3):251-66.

89. Suwan K, Hatthachote P, Panichkul S, Phromphetcharat V. Comparision of overweight and obesity in medical cadets before and after 6 months studying at Phramongkutklao College. J Med Assoc Thai. 2012; 95 Suppl 5:S142-8.

90. Westcott WL, Skaggs JM, Gibson JR, Annesi JJ, Reynolds RD, O'Dell JP. Comparison of two exercise protocols on fitness score improvement in poorly conditioned Air Force personnel. Percept Mot Skills. 2007; 104(2):629-36.

91. Patton JF, Vogel JA. Cross-sectional and longitudinal evaluations of an endurance training program. Med Sci Sports. 1977; 9(2):100-3.

92. Marniemi J, Dahlström S, Kvist M, SeppÄnen A, Hietanen E. Dependence of serum lipid and lecithin: cholesterol acyltransferase levels on physical training in young men. Eur J Appl Physiol Occup Physiol. 1982; 49(1):25-35.

93. Maleš B, Katić R, Ropac D. Development of aerobic endurance and repetitive strength in special army unit members. Coll Antropol. 1999; 23(2):723-8.

94. Mattila V, Tallroth K, Marttinen M, Ohrankammen O, Pihlajamaki H. DEXA body composition changes among 140 conscripts. Int J Sports Med. 2009; 30(5):348-53.

95. Wood PS, Krüger PE, Grant CC. DEXA-assessed regional body composition changes in young female military soldiers following 12-weeks of periodised training. Ergonomics. 2010; 53(4):537-47.

96. Grant CC, Mongwe L, van Rensburg DCJ, Fletcher L, Wood PS, Terblanche E, et al. The difference between exercise-induced autonomic and fitness changes measured after 12 and 20 weeks of medium-to-high intensity military training. J Strength Cond Res. 2016, 30(9):2453-9.

97. Yanovich R, Evans R, Israeli E, Constantini N, Sharvit N, Merkel D, Epstein Y, Moran DS. Differences in physical fitness of male and female recruits in gender-integrated army basic training. Med Sci Sports Exerc 2008, 40(11 Suppl):S654-659.

98. Loturco I, Ugrinowitsch C, Roschel H, Mellinger AL, Gomes F, Tricoli V, Gonzáles-Badillo JJ. Distinct temporal organizations of the strength-and power-training loads produce similar performance improvements. J Strength Cond Res. 2013, 27(1):188-194.

99. Sum C, Wang K, Choo D, Tan C, Fok A, Tan E. The effect of a 5-month supervised program of physical activity on anthropometric indices, fat-free mass, and resting energy expenditure in obese male military recruits. Metabolism 1994, 43(9):1148-1152.

100. Woodhead AB, 3rd, Moynihan ME. The effect of Aviation Officer Candidate's School on aerobic and anaerobic fitness. Mil Med 1994, 159(2):118-120.

101. Stacy RJ, Hungerford RL, McMahon BB. The effect of basic training on aerobic capacity and body fat in New Zealand army recruits. The New Zealand medical journal 1982, 95(722):876-878.

102. Powell GD, Dumitru D, Kennedy JJ. The effect of command emphasis and monthly physical training on Army physical fitness scores in a National Guard Unit. Mil Med 1993, 158(5):294-297.

103. Diment BC, Fortes MB, Greeves JP, Casey A, Costa RJ, Walters R, Walsh NP. Effect of daily mixed nutritional supplementation on immune indices in soldiers undertaking an 8-week arduous training programme. European journal of applied physiology 2012, 112(4):1411-1418.

104. Huang CJ, Kwok CF, Chou CH, Chou YC, Ho LT, Shih KC. The effect of exercise on lipid profiles and inflammatory markers in lean male adolescents: a prospective interventional study. Journal of investigative medicine : the official publication of the American Federation for Clinical Research 2015, 63(1):29-34.

105. Malavolti M, Battistini NC, Dugoni M, Bagni B, Bagni I, Pietrobelli A. Effect of intense military training on body composition. J Strength Cond Res. 2008, 22(2):503-508.

106. Katić R, Maleš B, Ropac D, Padovan M. Effect of programmed kinesiologic treatment on structural transformation of some strength and endurance manifestations in Croatian army draftees. Collegium antropologicum 2002, 26(1):229-237.

107. Bathalon GP, McGraw SM, Sharp MA, Williamson DA, Young AJ, Friedl KE. The effect of proposed improvements to the Army Weight Control Program on female soldiers. Mil Med 2006, 171(8):800-805.

108. Kraemer WJ, Mazzetti SA, Nindl BC, Gotshalk LA, Volek JS, Bush JA, Marx JO, Dohi K, GÓmez AL, Miles M. Effect of resistance training on women’s strength/power and occupational performances. Medicine & Science in Sports & Exercise 2001, 33(6):1011-1025.

109. Lester ME, Sharp MA, Werling WC, Walker LA, Cohen BS, Ruediger TM. Effect of specific short-term physical training on fitness measures in conditioned men. J Strength Cond Res. 2014; 28(3):679-88.

110. Cilliers J, Gordon N. Effect of the South African Army physical training instructor course on physical work capacity. S Afr J Sport Plrys Educ Recr. 1983; 6:3542.

111. Teyhen DS, Childs JD, Dugan JL, Wright AC, Sorge JA, Mello JL, et al. Effect of two different exercise regimens on trunk muscle morphometry and endurance in soldiers in training. Phys Ther. 2013; 93(9):1211-24.

112. Croteau KA, Young CJ. Effectiveness of a Navy remedial exercise intervention. Mil Med. 2000, 165(10):786-90.

113. Brock J, Legg S. The effects of 6 weeks training on the physical fitness of female recruits to the British army. Ergonomics. 1997; 40(3):400-11.

114. Lim CL, Lee L. The effects of 20 weeks basic military training program on body composition, VO_2max_ and aerobic fitness of obese recruits. J Sports Med Phys Fitness. 1994, 34(3):271-8.

115. Fortes MB, Diment BC, Greeves JP, Casey A, Izard R, Walsh NP. Effects of a daily mixed nutritional supplement on physical performance, body composition, and circulating anabolic hormones during 8 weeks of arduous military training. Appl Physiol Nutr Metab. 2011; 36(6):967-75.

116. Sporiš G, Harasin D, Bok D, Matika D, Vuleta D. Effects of a training program for special operations battalion on soldiers' fitness characteristics. J Strength Cond Res. 2012; 26(10):2872-82.

117. Marcinik EJ, Hodgdon JA, Vickers RR Jr.. The effects of an augmented and the standard recruit physical training program on fitness parameters. Aviat Space Environ Med. 1985;, 56(3):204-7.

118. Hickey JP, Donne B, O'Brien D. Effects of an eight week military training program on aerobic indices and psychomotor function. J R Army Med Corps. 2012; 158(1):41-6.

119. Williams AG. Effects of basic training in the British Army on regular and reserve army personnel. J Strength Cond Res. 2005; 19(2):254-9.

120. Legg SJ, Duggan A. The effects of basic training on aerobic fitness and muscular strength and endurance of British Army recruits. Ergonomics. 1996, 39(12):1403-18.

121. Williams AG, Rayson MP, Jones DA. Effects of basic training on material handling ability and physical fitness of British Army recruits. Ergonomics. 1999; 42(8):1114-24.

122. Kraemer WJ, Vescovi JD, Volek JS, Nindl BC, Newton RU, Patton JF, et al. Effects of concurrent resistance and aerobic training on load-bearing performance and the Army physical fitness test. Mil Med. 2004; 169(12):994-9.

123. Alemany JA, Nindl BC, Kellogg MD, Tharion WJ, Young AJ, Montain SJ. Effects of dietary protein content on IGF-I, testosterone, and body composition during 8 days of severe energy deficit and arduous physical activity. J Appl Physiol (1985). 2008; 105(1):58-64.

124. Shih KC, Janckila AJ, Kwok CF, Ho LT, Chou YC, Chao TY. Effects of exercise on insulin sensitivity, inflammatory cytokines, and serum tartrate-resistant acid phosphatase 5a in obese Chinese male adolescents. Metabolism. 2010; 59(1):144-51.

125. VIitasalo JT, Vainikka M. Effects of five-month conscription on physical fitness of 75 recruits. J Sports Med Phys Fitness. 1982; 22(1):95-101.

126. Hoiberg A. Effects of participation in the physical conditioning platoon. J Clin Psychol. 1978, 34(2):410-6.

127. Burke WP, Dyer F. Effects of Ranger Training on Selected Measures of Strength and Cardiovascular Fitness. In.: Army research inst for the behavioral and social sciences alexandria VA; 1980.

128. Hortobagyi T, Katch FI, Lachance PF. Effects of simultaneous training for strength and endurance on upper and lower body strength and running performance. J Sports Med Phys Fitness. 1991; 31(1):20-30.

129. Sewani-Rusike CR, Mudambo KS, Tendaupenyu G, Dzuda C, Tafirenyika A, Zenda E. Effects of the Zimbabwe Defence Forces training programme on body composition and reproductive hormones in male army recruits. Cent Afr J Med. 2000; 46(2):27-31.

130. Sporiš G, Harasin D, Baić M, Krističević T, Krakan I, Milanović Z, et al. Effects of two different 5 weeks training programs on the physical fitness of military recruits. Coll Antropol. 2014; 38(Supplement 2):157-64.

131. Harman EA, Gutekunst DJ, Frykman PN, Nindl BC, Alemany JA, Mello RP, et al. Effects of two different eight-week training programs on military physical performance. J Strength Cond Res. 2008; 22(2):524-34.

132. Kraemer WJ, Vogel JA, Patton JF, Dziados JE, Reynolds KL. The effects of various physical training programs on short duration, high intensity load bearing performance and the Army physical fitness test. In.: Army research inst of environmental medicine natick MA; 1987.

133. Morel DS, Moreira-Marconi E, Neto SBS, Domingos LLP, de Souza PL, de Sá Caputo DdC, et al. Effects of whole body vibration intervention on handgrip strength of Brazilian healthy soldiers. Afr J Tradit Complement Altern Med. 2017;14(4 Suppl):28-32.

134. Knapik J, Darakjy S, Scott SJ, Hauret KG,Canada S, Marin R, et al. Evaluation of a standardized physical training program for basic combat training. J Strength Cond Res. 2005; 19(2):246-53.

135. Reedy JD. Evaluation of the effectiveness of the physical training program during the basic infantry training cycle. In.: Army medical research lab fort KNOX KY; 1954.

136. Allen C, O'Hara W, Myles W, Townshed R, Brown T. An Evaluation of the Physical Training of Officer Candidates at Canadian Forces Officer Candidate School. In.: Defence and civil INST of environmental medicine downsview (ontario); 1977.

137. Cederberg H, Mikkola I, Jokelainen J, Laakso M, Härkönen P, Ikäheimo T, et al. Exercise during military training improves cardiovascular risk factors in young men. Atherosclerosis. 2011; 216(2):489-95.

138. Mayo MJ, Grantham JR, Balasekaran G. Exercise-induced weight loss preferentially reduces abdominal fat. Med Sci Sports Exerc. 2003; 35(2):207-13.

139. Lieberman HR, Kellogg MD, Bathalon GP. Female marine recruit training: mood, body composition, and biochemical changes. Med Sci Sports Exerc. 2008; 40(11 Suppl):S671-6.

140. Marcinik EJ, Hodgdon JA, O'Brien JJ, Mittleman K. Fitness changes of Naval women following aerobic based programs featuring calisthenic or circuit weight training exercises. Eur J Appl Physiol Occup Physiol. 1985; 54(3):244-9.

141. Bingham CM, Lahti-Koski M, Absetz P, Puukka P, Kinnunen M, Pihlajamäki H, et al. Food choices and health during military service: increases in sugar-and fibre-containing foods and changes in anthropometric and clinical risk factors. Public Health Nutr. 2012; 15(7):1248-55.

142. Lee L, Kumar S, Leong LC. The impact of five-month basic military training on the body weight and body fat of 197 moderately to severely obese Singaporean males aged 17 to 19 years. Int J Obes Relat Metab Disord. 1994; 18(2):105-9.

143. Rosendal L, Langberg H, Skov-Jensen A, Kjaer M. Incidence of injury and physical performance adaptations during military training. Clin J Sport Med. 2003; 13(3):157-63.

144. Dias I, de Salles BF, Novaes J, Costa PB, Simão R. Influence of exercise order on maximum strength in untrained young men. J Sci Med Sport. 2010; 13(1):65-9.

145. Tai-Min L, Chi-Ting C, Su-Sing L, For-Wey L. The influence of recruitment training on serum lipid shifts. Mil Med. 2004; 169(12):1011-3.

146. Knapik JJ, Wright JE, Kowal DM, Vogel JA. The influence of U.S. Army Basic Initial Entry Training on the muscular strength of men and women. Aviat Space Environ Med. 1980; 51(10):1086-90.

147. Schlessinger BS. Influences of exercise and diet on the blood lipids of military population. Mil Med. 1958; 123(4):274-8.

148. Mousavinasab F, Tahtinen T, Jokelainen J, Koskela P, Vanhala M, Oikarinen J, et al. Lack of increase of serum adiponectin concentrations with a moderate weight loss during six months on a high-caloric diet in military service among a young male Finnish population. Endocrine. 2005; 26(1):65-9.

149. Friedl KE, Moore RJ, Martinez-Lopez LE, Vogel JA, Askew E, Marchitelli L, et al. Lower limit of body fat in healthy active men. J Appl Physiol (1985). 1994; 77(2):933-40.

150. Lichton IJ, Miyamura JB, McNutt SW. Nutritional evaluation of soldiers subsisting on meal, ready-to-eat operational rations for an extended period: body measurements, hydration, and blood nutrients. Am J Clin Nutr. 1988; 48(1):30-7.

151. Teves MA, Wright JE, Vogel JA. Performance on selected candidate screening test procedures before and after Army basic and advanced individual training. In.: Army medical research and development command fort detrick MD; 1985.

152. Mikkola I, Jokelainen JJ, Timonen MJ, Harkonen PK, Saastamoinen E, Laakso MA, et al. Physical activity and body composition changes during military service. Med Sci Sports Exerc. 2009; 41(9):1735-42.

153. Crawley AA, Sherman RA, Crawley WR, Cosio-Lima LM. Physical fitness of police academy cadets: Baseline characteristics and changes during a 16-week academy. J Strength Cond Res. 2016; 30(5):1416-24.

154. Lonsdale D. Physical fitness program in flight cadets during basic training at primary training school in the RCAF. Med Serv J, Can. 1960; 16:792-800.

155. Bartlett CG, Stankorb S. Physical performance and attrition among U.S. Air Force trainees participating in the basic military training fueling initiative. Mil Med. 2017, 182(1-2):e1603-9.

156. Guezennec CY, Satabin P, Legrand H, Bigard AX. Physical performance and metabolic changes induced by combined prolonged exercise and different energy intakes in humans. Eur J Appl Physiol Occup Physiol. 1994, 68(6):525-30.

157. Maloney JP, Cheney R, Spring W, Kanusky J. The physiologic and psychological effects of a 5-week and a 16-week physical fitness program. Mil Med. 1986, 151(8):426-33.

158. Daniels WL, Kowal DM, Vogel JA, Stauffer RM. Physiological effects of a military training program on male and female cadets. Aviat Space Environ Med. 1979, 50(6):562-6.

159. Chai LY, Ong KC, Kee A, Earnest A, Lim FC, Wong JC. A prospective cohort study on the impact of a modified Basic Military Training (mBMT) programme based on pre-enlistment fitness stratification amongst Asian military enlistees. Ann Acad Med Singapore. 2009; 38(10):862-8.

160. Kowal DM, Patton JF, Vogel JA. Psychological states and aerobic fitness of male and female recruits before and after basic training. Aviat Space Environ Med. 1978; 49(4):603-6.

161. Hagnäs MP, Cederberg H, Mikkola I, Ikäheimo TM, Jokelainen J, Laakso M, et al. Reduction in metabolic syndrome among obese young men is associated with exercise-induced body composition changes during military service. Diabetes Res Clin Pract. 2012; 98(2):312-9.

162. Williams AG, Rayson MP, Jones DA. Resistance training and the enhancement of the gains in material-handling ability and physical fitness of British Army recruits during basic training. Ergonomics. 2002; 45(4):267-79.

163. Gordon NF, Van Rensburg JP, Moolman J, Krüger PE, Russell HM, Grobler HC, et al. The South African Defence Force physical training programme. Part I. Effect of 1 year's military training on endurance fitness. S Afr Med J. 1986, 69(8):477-82.

164. Gordon NF, Moolman J, Van Rensburg JP, Russell HM, Krüger PE, Grobler HC, et al. The South African Defence Force physical training programme. Part II. Effect of 1 year's military training on muscular strength, power, power-endurance, speed and flexibility. S Afr Med J. 1986; 69(8):483-90.

165. Cederberg H, Rajala U, Koivisto VM, Jokelainen J, Surcel HM, Keinänen-Kiukaanniemi S, et al. Unacylated ghrelin is associated with changes in body composition and body fat distribution during long-term exercise intervention. Eur J Endocrinol. 2011; 165(2):243-8.

166. Cederberg H, Koivisto VM, Jokelainen J, Surcel HM, Keinänen-Kiukaanniemi S, Rajala U. Unacylated ghrelin is associated with changes in insulin sensitivity and lipid profile during an exercise intervention. Clin Endocrinol (Oxf). 2012; 76(1):39-45.

167. Cocke C, Dawes J, Orr RM. The use of 2 conditioning programs and the fitness characteristics of police academy cadets. J Athl Train. 2016; 51(11):887-96.

168. Nascimento CL, Constantini AC, Mourão LF. Vocal effects in military students submitted to an intense recruit training: a pilot study. J Voice 2016; 30(1):61-9.

169. Himashree G, Mohan L, Singh Y. Yoga practice improves physiological and biochemical status at high altitudes: a prospective case-control study. Altern Ther Health Med. 2016; 22(5):53-9.
