# Supplementary material for: Dietary, physical activity, and weight management interventions among active-duty military personnel: a systematic review
Source: Mil Med Res. 2018 Dec 24;5:43. doi: 10.1186/s40779-018-0190-5 (PMC6309065; doi:10.1186/s40779-018-0190-5)
Supplement: Supplementary file 2 — Full description of included interventions, outcome measurements, results in addition to sample size, age, setting and quality rate. (DOCX 100 kb) [file 40779_2018_190_MOESM2_ESM.docx]

Additional file 2: Full deception of included interventions, outcome measurements, results in addition to sample size, age, setting and quality rate

| Study No. | Author  Study design  Total quality rate | Number,  Age,  Profession | Country/setting | Intervention and/or control,  (tailored or generic advice),  Curriculum used (yes/No) | Duration of the intervention,  No. of contacts  Mode of delivery,  Follow-up | Main outcomes,  Summary of results |  |
| --- | --- | --- | --- | --- | --- | --- | --- |
| **1** | - Dennis et al, 1999[21] - RCT - Strong | - 39 - 31±6 years - Navy | Service members of US aircraft carrier | - The program includes diet; behavior modification; cognitive, emotional, and social factors that influence weight management; and exercise - Used a standardized instructor manual - Control: Usual care: nutrition fact sheets and brochures if requested in addition to Level I program of exercise | - 6 months - Weekly sessions - Small group lectures Participant received a notebook of course material - 6 months | - Weight, BMI and BF% - Weight and BF% was significantly reduced at 6-month follow-up (8.6±5 kg of original weight and 7.8%±3.5% of original BF) in comparison to the control group - The effect size for weight using d ppc2 = -0.26 |  |
| **2** | - Shrestha et al, 2013[35] - RCT - Weak | - 28.7 ± 4.4 in the control group and 35.0 ± 8.7 - 28-35 years - Soldiers | Walter Reed Health Care System (US) | - The intervention group received accelerometers to track activity levels online over time and modify exercise routines as desired. Participants received 1.5 hours of standardized health instruction - Used a standardized instructor manual - Usual care: self-directed exercise and/or mandated physical training | - 6 months - N/A - Internet: soldiers have access to website where they can track their records - 2, 4, and 6 months | - Weight, BMI, BF%, WC, resting heart rate and total IPAQ (MET-minutes/week) - No significant difference of any outcome measurements at 6 months follow-up in comparison of the control group |  |
| **3** | - Earles et al, 2007[29] - Retrospective study - Moderate | - 387 - 29.57 years - Soldiers | US military in Hawaii | - The intervention started as a one-week inpatient intervention (Phase I) with 12 months of weekly outpatient follow-up (Phase II). Only in Phase I were participants offered orlistat+O26. The curriculum included sessions on nutrition and physical fitness such as meal planning and basic nutrition - Used a standardized instructor manual - No control group | - One year - One week day treatment followed by weekly follow-up for 12 months - Classes/sessions during phase one follow-up by one to one counseling - 12 months | - BMI and percentage weight loss - BMI was significantly reduced after 12 months' follow-up. - Both groups lowered their BMIs after one year with no significant difference between those who used orlistat and those who did not. - The percentage weight loss was 6-7% and BMI reduced from 31.97 to 29.11 |  |
| **4** | - Hunter et al, 2007[28] - Two-group randomized controlled - Strong | - 450 - 33.5±7.4 in the intervention group and 34.4±7.2 in the control group - Soldiers |  | - The intervention received orientation on calculating calories and energy expenditure. Participants were instructed to monitor their weight weekly through a weight-tracking chart by submitting electronic food and exercise diaries. Participants received weekly online lessons and feedback from a counselor and two brief motivational interviews by telephone - Used individualized feedback - Usual care: members attend one annual assessment for diet and weight with access to fitness center, weight loss and cooking classes, nutrition consultations, and individual fitness assessments | - 6 months - Orientation session with weekly lessons and individualized counseling feedback from a counselor online till 6 months in addition to two brief motivational telephone calls interviewing - One to one online counseling with motivational interviewing telephone calls and online diaries, feedback and lessons - 6 months | - Weight, percentage weight loss, BMI, BF %, WC, meat and snacks screener, fruit, vegetables, bean screener and total IPAQ - There were significant improvement in weight, percentage weight loss, BMI, BF % and waist in the intervention in comparison to usual care group. The change in weight, BMI, WC, BF% was -1.3, -0.5, -2.1, -0.4 respectively. There was significant improvement for meat and snacks screener (-5.2 score change), fruit, vegetables, bean screener (2.3 score change) in the BIT group in comparison to the control. There was no significant difference of total IPAQ between the two groups - Effect size for weight difference between groups (Cohen’s d) = -0.178 |  |
| **5** | - Smith et al, 2010[33] - Two-group randomized design - Weak | - 46 - 27.5±7.3 and 28.9±7.5 years in both groups - Soldiers | US army | - There are two arms: (1) weigh-to-stay which had three educational sessions: nutrition, exercise, and an individualized counseling and (2) weigh-to-stay with meal replacement. Participants were provided with extra calorie-controlled meal plans - Tailored and generic - No control group | - Around 5 months - three educational sessions typically completed within a 2-week - Educational classes, private counseling (one to one) and brochure - Around 5 months follow up | - Weight, BMI, BF %, fat mass and lean mass - Meal replacer group demonstrate significant loss in weight, body fat percentage and fat mass in comparison with weigh to stay. - Both treatment groups achieved significant reduction in comparison to baseline for weight, body fat % (-1.9% in the meal replacer group) and fat mass. Weight loss was around -3 kg - Effect size of weight loss using d ppc2= -0.006 |  |
| **6** | - James et al, 2001[26] - Longitudinal study - Weak | - 48 - 27.7 and 31.7 in both groups - Navy | U.S. Navy and Army personnel | - Three weeks inpatient intervention with 12 months of weekly outpatient follow-up. There are two intervention groups: one group received weekly guidance and monitoring at the hospital and the other group received guidance through interactive video and Web-page (IATV) - No control group | - 12 months - 3 week daily treatment with weekly follow-up for 12 months - In patient session, interactive video link-ups and group support - 3 months follow-up | - BMI, weight loss and percentage weight loss - No significant difference was found in BMI between the two groups at 3 months follow up. Significant decrease in all variables were found in comparison to baseline |  |
| **7** | - Shay et al, 2009[32] - Three group Randomize design - Weak | - 39 - 35 years - Navy | Large east coast naval hospital in the US | - The intervention has 3 arms according to the self-monitoring method: (1) paper diary, (2) web-based diary and (3) PDA diary. Each participant was given instructions on how to record their food, beverage, and exercise using their assigned diary - Generic - No control group | - 12 weeks - Weekly sessions for 8 weeks. Participants are asked to record in their diaries for 12 weeks - Sessions/lectures - 6 weeks, 12 weeks | - Weight, BF % and WC - There was no significant interaction between diary preference and time for any of the three variables (weight, % BF, and WC). Subjects as a whole demonstrated a significant decrease in weight (−2.8 kg., *P<*.001), WC (−2.2 in., *P<*.001) and estimated percent body fat (−1.6%, *P<*.001). Comparing those who used the diary they preferred and those who did not, there was no significant for a difference between groups on change in 3 variables at week 6 and 12. |  |
| **8** | - Simpson et al, 2004 [27] - Retrospective study - Weak | - 65 - 28 years - Navy | US army | - The LE3AN Program consists of two phases. Phase I is a one-week intensive outpatient treatment. Phase II consists of weekly follow-up visits (60 minutes each) for a year that assists the participants in sustaining lifestyle changes - Tailored and generic - Used a standardized instructor manual - No control group | - 1 year - One week day treatment followed by weekly follow-up for 12 months - Lectures and exercise classes - 6 month follow up | - Weight - There was a significant mean weight loss after 6 months (mean weight loss=12.12 pounds). |  |
| **9** | - Robbins et al, 2006[17] - NRCT - Moderate | - 68,591 - 30.4 and 31.9 years in both groups - Air force | US Air force bases | - Self-directed behavior change booklets with weekly e-mail messages were used to educate, motivate, and sustain behavior change among participants - Generic - Curriculum used - Placebo: controls are ADAF members meeting the BMI criteria at other USAF bases during the same period. | - 1 year - Two booklets and 52 weekly e-mails (one year) - Self-help booklet plus educational email - 12 months | - Weight - Men and women had a significant reduction in mean weight in comparison to the control group after 12 months (-1.3lb for men and -1.0lb for women). |  |
| **10** | - Mantzios et al,2014 [51] - RCT - Moderate | - 63 - 21-22 years - Soldiers | Military base in Greece | - The intervention is based mainly on mindfulness and meditation training with two arms: (1) mindfulness meditation group which received meditation on helpful topics for losing weight (2) the mindfulness with self-compassion group received the same meditation training, with an additional hour of meditation training covering loving kindness meditation - Generic - Curriculum used - Usual care: only received training on diet-related topics for losing weight such as the automaticity of eating and having smaller plates to encourage eating less. | - 5 weeks - 2-3 day group sessions with daily practice sessions over the first 5 week - Group sessions plus written psycho-educational material - 5 weeks, 6 and 12 months | - Weight change and cumulative weight change. At the 5-week measurements, the mindful group did not significantly differ from the mindfulness group, but both experimental groups were significantly different from the control group. At the 6-month, the mindful group lost significantly more weight than both the mindfulness and control groups, while the latter groups were also significantly different from each other. At the 1-year follow-up, all groups were not significantly different from each. - The cumulative mean loss was 1.8 and 3 kg for mindfulness meditation group and mindfulness with self-compassion group respectively. | |
| **11** | - Bowles et al, 2006[28] - Pretest posttest study - Weak | - N/A - N/A - Multiple branches: Navy, Air force, Marine Corps. | US army | - The intervention consisted of 1-week, intensive (phase 1) outpatient program with 1 year of follow-up treatment (phase 2) which focused on relapse prevention. Participants were trained on goal setting and many nutritional topics - Seems to be generic - There is outline for phase 1 of the intervention - No control group | - N/A - The first week includes one week every day training with one year follow-up treatment (average 18 sessions) - mainly group sessions - one month, 6 month and one year | - Weight loss change and BMI - Weight and BMI were significantly decreased for all participants (men and women) after one year. - Weight loss was 11 pounds for men and 14 pounds for women. |  |
| **12** | - Daniels et al, 1980[21] - Pretest posttest study - Weak | - 18 - N/A - Conscripts | Military academy in the US | - Physical training consisted of calisthenics, grass drills and a 30-minute run 5-6 times per week. All cadets were required to participate in either sport and to attend physical education classes - No control group | - 2 years - 5-6 times of physical training per week - N/A - End of first summer training, beginning and end of Second summer training and end of second year training | - Weight, BF%, VO_2_ max, HR, maximal voluntary isometric contractions (to assess strength for legs, arms, shoulders and trunk), minute ventilation - VO_2_ increased significantly over the two-year period for males. Minute ventilation increased and remained elevated throughout the second academic year. Females had significant increase in VO_2_ max. HR max and minute ventilation changed significantly for females. Over the summer of first year, males only showed significant increase in leg extensor strength. Males showed larger increases in upper body strength and upper torso. |  |
| **13** | - McDoniel et al, 2008[31] - quasi-experimental control trial - Moderate | - 54 - 28.0 ± 7.3 years - Airforce | US Air Force | - Participants received an educational handout. A personalized caloric goal was established by reducing 250–500 kcal/day based on hand-held indirect calorimeter. All participants participated in the four sessions on basic nutrition topics - Tailored - Curriculum used - Usual care: Usual care participants received a nutrition program based on estimated energy expenditure | - 90 days - N/A - Sessions - 3 months | - Weight, BMI, BF %, Cholesterol (mg), carbohydrates %, protein % and fat% - The experimental group lost significantly more weight than the control groups at 3-month follow-up (-4.3±3.3 vs -1.8±3 kg respectively). - Effect size (Cohen's d) =0.124 - There was a significant reduction in total calorie intake (-729 ± 1047) and reduction in dietary cholesterol levels (-62.9±217.2 mg/day) in comparison to baseline after 3 months. - No difference in percentage fat intake. |  |
| **14** | - Sammito et al, 2013[49] - Pretest posttest study - Weak | - 665 - 40 ± 9.4 years - Soldiers | German Military Forces | - Individual advice for doing more daily physical activity and encouraging sports activities such as Nordic walking and swimming. Nutritional counseling was provided to reduce fat intake and increase consumption of fruit and vegetables - No control group | - N/A - N/A - One to one counseling - 3, 6, 9, 12, 18 and 24 month | - Weight, BMI, WC & physical capacity - Significant improvement in all outcomes at 3, 6, 9, 12, 18 and 24 month follow up. The total weight and WC reduction was around 3.2 kg and 3.1 cm respectively. |  |
| **15** | - Smith et al, 2012[36] - RCT - Weak | - 435 - N/A - Soldiers | Womack Army Medical Center | - Participants received Weigh-to-stay program plus 1-month supply of Orlistat 60 mg at each monthly visit. Participants received the educational program which contained information about weight loss, healthy eating and exercise, energy and fat content of foods with multivitamins - Tailored and generic - Standard care: Weigh-to-Stay program which consisted of nutrition, exercise, and individualized educational sessions | - 6 months - Three education sessions (30 minutes to 2-hour). - Individual advice + educational materials + educational sessions (classes) - 6 month | - Weight, BMI, CIRC and DEXA body fat %, fat mass and lean mass Energy intake (kcal/d), protein, carbohydrate, fat intake (% energy intake), energy expenditure (kcal/d) - In comparison to the control group, the Orlistat group reduced body fat % (-2.2±3.6 vs -0.6± 1.8) and fat mass (-2.5±3.9 vs -1.3±2.5) more than the placebo group, and lost less fat-free mass. Both showed significant reduction in weight, BMI, CIRC body fat %, fat mass and lean mass in comparison to baseline. The total weight reduction for the intervention and control groups was -3.8±3.5 and -2.7±4.4 kg respectively) - Effect size for weight reduction dppc2 =-0.072 - No significant differences in energy intake or diet composition including fat intake between groups over time - No significant within or between group changes for energy expenditure were observed |  |
| **16** | - Trent et al, 1993[22] - Cohort study - Moderate | - 624 - 30 years - Navy | US Navy | - The intervention contained 3 levels of command. Level I is the basic remedial conditioning program consisting of supervised group exercise sessions, and may include nutrition and behavior modification techniques. Level II is more intensive and contains 80 hours of weight-management counseling. Level III programs entail 6 weeks of inpatient therapy with trained counselors and medical supervision - Guideline used - No control group | - 6 months for level 1 & around 6 weeks for level 2 and 3 - 3-4 times per week for level 1 & 80 hours for level 2 - group sessions - 6 week, 6 month and 1 year | - Weight, BMI, BF % and lean mass - There was significant reduction in BF% (-3.8%), weight (-1.9 lbs) and BMI (-1.2) for both men and women at 1 year follow up. Lean body mass (3.3 lbs) significantly increased for both sexes. Level III was found to be more effective than both Level I and Level II in reducing body fat % and the percentage of obese people |  |
| **17** | - GAMBERA et al, 1995[23] - RCT - Weak | - 32 - 32.7 ± 7 and 33.3 ± 6 years for both groups - Air force | US Air Force | - There are 2 interventions: (1) the exercise group which received sessions incorporating the use of large muscle groups at an intensity of 60% to 80% of maximum heart rate for 40 minutes; (2) the exercise plus diet group received weekly individualized dietary counseling - No control group | - 90 days - 3 times per week for the exercise program - individualized dietary counseling - 90 days | - Weight, BMI, VO_2_ max, energy/calories per day, cholesterol intake (mg), carbohydrates %, protein %, total fat %, saturated fat %, fibers intake and serving per day for fruit, vegetables - In the exercise plus diet group, energy intake from fat (39% - 23%), total energy intake, saturated fat intake, cholesterol intake were reduced significantly for both men and women after 3 months. 2) Carbohydrates intake, dietary fibers (71%), serving of fruit (0.8-3.6) and vegetables (2-2.6) were significantly increased for in the intervention group - The exercise plus diet group showed significant weight (-1.3 kg for men and -2.8 kg for women in the treatment group) and BMI reduction in comparison to exercise only group for both men and women without significant differences between both sexes - Both groups improved VO_2_ max (14% and 38% increase in the exercise only and exercise plus diet group) |  |
| **18** | - Herzman-Harari *et al.*, 2013[56] - Pretest posttest study - Weak | - 44 - 18.8 ± 0.1 years - Conscripts | Israeli Military | - The intervention included 15 sessions that were presented in many formats such as lectures, interactive activities on nutritional topics, and accompaniment to various meals in the mess hall to provide guidance for making informed food choices - No control group | - 2 months - 15 sessions over 2 months - Lectures, posters, interactive activities, discussions, film screenings and handouts - 2 months and 4 months after the intervention | - Weight, BMI. Energy (kcal), protein %, Fat %, Carbs % and Dietary fibers (g) - Average BMI increased significantly with average 1.9 kg weight gain - There was significant improvement in total energy, protein %, fat %, carbohydrate % (4% reduction) and dietary fibers (9.2 g increase). A low significant increase of fruit and vegetables serving per day. {0.74 ± 0.25 servings per day (0.3 servings of fruit and 0.44 servings of vegetables)} |  |
| **19** | - Hofstetter et al, 2012[47] - NRCT - Weak | - 259 - 20.69 ± 6 1.17 years - Conscripts | Swiss Military school | - The intervention group had a weekly outdoor circuit training program in addition to the standard physical training program. A physical education teacher instructed recruits on how to perform the specific training exercises. - A standardized protocol was used - Standard physical training which includes strength and aerobic fitness training. | - 7 weeks - 2 sessions per week for the standard training and one additional session (60 minutes) for the intervention group - Group sessions - 7 weeks | - Weight, BMI, standing long jump (m), Seated 2-kg shot put (m), One-leg standing test (s), Trunk muscle strength test (s), Progressive endurance run (min) - A significant improvement in 1-Leg standing test (18.44 ± 21.88 increase), trunk muscle strength test (40.26 ± 58.20 increase), progressive endurance (1.95 ± 2.62), Swiss physical fitness test and battery overall score (8.87 ± 8.58 increase) compared to baseline for both groups with greater improvements in the intervention in all variables except endurance compared to the control group - No significant difference in weight and BMI between the two groups |  |
| **20** | - Glick and Kaufmann, 1975[55] - Pretest-posttest study - Weak | - 129 - N/A - Conscripts | N/A | - Intensive physical training in addition to 1-2 hours per week of physical education. Subjects were eating ad libitum diet. - No control group | - 12 weeks - N/A - N/A - 12 weeks (post intervention) | - Weight and Sum of skinfold skin thickness (SSFT) - There was significant weight gain around 1.1 kg with significant increase of sum skinfold skin thickness (SSFT) (around 1.2 mm) - SSFT (+1.2) and weight (+1.1) was increased significantly in the group with initial small SSFT and weight (-2.9 kg) and SSFT (-10.6 mm) decreased significantly with large initial SSFT |  |
| **21** | - Sammito *et al.*, 2016 [53] - Retrospective study - Weak | - 334 - 43.5±6.9 years - Soldiers | Military Sport School of the German Armed Forces | - The intervention includes lessons on nutrition, sport exercise and lifestyle factors. Individual nutrition consultation was given according to the recommendations of the German Society of Nutrition. - Both generic and tailored advice. - No control group | - 3 weeks course at the beginning with another one week course at month 12 and 24 in addition to 12 month follow-up consultation - N/A - N/A - 6, 12, 18 and 24 month | - Body weight, BMI and WC - Body weight (-3.4 ± 6.6 kg), BMI, WC (-3.8 ± 6.4 cm) showed significant improvement in all follow-up dates of intervention |  |
| **22** | - Tomczak *et al.*, 2016 [54] - Pretest posttest study - Weak | - 60 - N/A - Soldiers | Polish army | - Obligatory physical education classes that take from 2 to 4 h per week (total 200 hours during 9 months) in addition to physical training - No control group | - 9 months - Physical education classes that take from 2 to 4 h per week in addition to physical Training - Classes - 9 months | - Weight, BMI, lean body mass, push-ups, sit-ups, 1000 m run and long jump (cm) - There was statistically significant increase of the BMI, weight (70.7 to 72.8 kg) and lean mass (60.5 to 62.1 kg) after 9 months - There was significant improvement of 1000 m run (43.3 to 48.6 points) |  |
| **23** | - Reppart *et al.*, 1978[40] - Longitudinal study - Weak | - 160 - N/A - Air force | USA Air Force | - Treatment A: Patients were given copies of dietary programs with minimal support. Treatment B: 30 min. appointment with a dietitian with one hour weekly behavioral modification class - No control group | - 3 months - Weekly sessions for treatment B - Classes plus one to one session - 1, 2, 3, 4 month | - Weight - Treatment B participants achieved significant weight loss after 2 and 4 months in comparison to treatment A |  |
| **24** | - James *et al.*, 1999[24] - Longitudinal study - Weak | - 112 - 32 years - Soldiers | US army | - The LE3AN Program consisted of three-week intensive in-patient treatment, then one week of daily treatment followed by weekly follow-up visits. - A standardized protocol was used - Both generic and tailored - No control group | - 1 year - Two week day inpatient hospitalization followed by weekly follow-up for 12 months - lectures and exercise classes - 6, 12, 18 | - Weight - There were significant weight reduction at 12 (18% weight loss) and 18 months (13% weight loss) follow up |  |
| **25** | - Davis 1996[37] - Longitudinal study - Weak | - 125 - 26 years - Soldiers | US army hospital in Germany | - The program consisted of counseling by a dietitian, then three-week in-patient treatment, then outpatient program focused on relapse prevention - Both generic and tailored - No control group | - N/ N/A - N/A - one to one and group counseling - 3 weeks and 6 months | - Weight and BF% - There was significant reduction in weight (-2.4 pounds) and BF% (-3.8%) after 6 months |  |
| **26** | - Veverka *et al.*, 2003[41] - RCT - Moderate | - 42 - 30-44 years - Air force | US Air Force | - Those in the treatment group were required to visit the website at least once each month to view the messages for reviewing information on diet and exercise. Subjects received the appropriately tailored newsletter content (one for diet, one for physical activity) - A standardized protocol was used - Both generic and tailored - control group (no access to Web site) | - 6 months - N/A - Internet and printed material - 6 months | - Weight, BF%, BMI, waist to hip ratio and VO_2_ score - There was significant reduction in weight (-2.2 kg), BMI, waist to hip ratio and body fat (-1.5%) at posttest with no significant improvement in VO_2_ score. - Effect size Cohen's d = 0.381956 |  |
| **27** | - Bingham et al, 2012[52] - NRCT - Weak | - 604 - 18-21* years - Conscripts | 2 garrisons in Finland | - Intervention: training workshops were conducted for the cafeteria personnel to promote healthy food supply and to reach the nutritional goals of the intervention - Control: no intervention (historical controls performing military service one year before the intervention group). | - 2 years - Around 6 workshops - Lectures and training workshops for cooks, supervisors and catering staff - 8 weeks and 6 months of service | - Cereal index, fruit and vegetables index, fat index and sugar index - Cereal index showed significant change at 6 months (3.3 vs 2.98) and fat index was significantly lower at 8 weeks (0.55 vs 0.68) in comparison to the control group. No significant change in fruit and vegetable intake. |  |
| **28** | - Uglem et al, 2014[50] - NRCT - Weak | - 479 - 19.7 ± 2.8 for the intervention and 19.2 ± 1.3 for the control groups - Conscripts | Two military camps in Norway | - The intervention was directed to the kitchen personnel by increasing the availability of vegetables and semi-whole grain bread, with nutrition information through posters, brochures, and folders. Cooks were trained to prepare new and healthy dishes - Generic - Placebo | - 5 months - N/A - Training workshops and printed materials such as posters and leaflets - 5 months | - Intake (grams per day) of vegetables, fruits, and semi whole grain bread. All intervention groups have significantly more intake of vegetables, fruits, and semi-whole grain bread after 5 months in comparison to the control. - Those in the low and medium intake groups at baseline had the highest percentage increase, while the group with high intake at baseline had no significant increase in any outcomes in comparison to baseline. |  |
| **29** | - Fiedler et al, 1999[25] - RCT - Weak | - 824 - N/A - Conscripts | US Air force | - The intervention (healthy fare) is characterized by providing two 3-week menu plans with three daily meals. The menu supplies 30% calories from fat, with high fiber and calcium choices, no fried food and fat-free salad dressing. There is daily walking and physical conditioning training - Curriculum used - Standard care (traditional fare). The control group had the same nutritional curriculum offered during their academic classes, with the same physical conditioning program | - 6 weeks - N/A - N/A - N/A | - BMI, overall Diet Quality Index (DQI), percentage of Calories from total fat, bread, cereal, and legume Intake, fruit and vegetable Intake, protein intake and caloric intake - Improvement in the DQI for those who ate the healthy fare almost doubled, with only slight improvement for the group consuming the traditional fare. Calories from total Fat decreased to 19% for the healthy fare and increased to 37% for the traditional fare. Carbohydrate intake increased for the healthy group from 3.7 to 4.4 servings and decreased for the traditional group from 3.4 to 3.0 servings Fruit and vegetable intake decreased for both groups, but was slightly higher for the healthy group. There were no significant differences between the two groups in BMI. |  |
| **30** | - Crombie et al, 2013 [37] - RCT - Moderate | - 458 - 27 years - Soldiers | US Air Force | - Participants received an educational handout. A personalized caloric goal was established by reducing 250–500 kcal/day based on hand-held indirect calorimeter. All participants participated in the four sessions on basic nutrition topics - Usual care: Usual care participants received a nutrition program based on estimated energy expenditure | - One year - N/A - Posters - 6 months and 1 year | - Total energy, percent of total energy from fat, percent of total energy from saturated fat and number of servings of fruit, vegetables, and whole grains. - The intervention group consumed lower energy intake (-116±31 kcal), total fat (-3.6%±0.9%), saturated fat (-0.9%±0.2%), discretionary fats and refined grains (-0.5±0.2 1-oz equivalents) in comparison to control at 6-months' follow-up. - Only refined grain reduced significantly in comparison to control at 6 and 12 months' follow-up (2.3±1.7 g vs 2.8±2.4 g; and 2.4±1.8 g vs 2.6±1.8 g; respectively). All outcomes improved at 12 months' follow-up in comparison to baseline. - No differences were observed in intake of whole grains, fruit and vegetables between the intervention and control groups at 6 and 12 months' follow-up. |  |
| **31** | - Thorsen et al,2010[45] - Pretest posttest study - Weak | - 190 - over 40 years - Soldiers | Military base in Denmark | - The intervention used a participatory and empowering approach, self-monitoring and networking among the canteen staff to increase fruit & vegetables for lunch, making environmental changes in the canteens by giving access to tasteful and healthy food choices. - No control group | - 6 days - N/A - N/A - 1 year and 5 years | - Fruit & Vegetables consumption (g/meal/customer) - At the military base canteen, there was insignificant increase of 18 g of fruit and vegetables per customer per day compared to the baseline at 5-year follow-up. There was significant increase of fruit and vegetables per customer per day after 1 year |  |
| **32** | - Buffington et al, 2016[42] - RCT - Weak | - 153 - N/A - Air Force | U.S. Air Force Academy’s (USAFA) | - The intervention contains 2 arms: (a) the COPE intervention alone (E2), individuals were taught how to monitor stressful events and how to change negative beliefs into positive ones. Also, they were taught problem-solving and coping skills; (b) the combined energy balance educational intervention (E1) with the COPE CBT-based intervention. The energy balance educational intervention was knowledge-based and included topics such as the energy balance equation, metabolic rate and making healthy choices. - Seems generic - Standard care: received one military training session as attention control per week. | - 12 weeks - 10 sessions during 12 weeks/one per week - Slide-based lecture and emails - 12 weeks (post intervention) | - Percentage kcal of carbs, protein, fat, saturated fat and BF %. - The combined intervention group made more favorable dietary choices as there was statistically significant improvement of carbohydrate intake. Significant decrease in total fat intake in the combined group. There was a significant decrease in saturated fat intake in the 2 intervention groups (7.63 vs 10.31% in E1 and 7.55 vs 10.14% in E2) - Both experimental groups demonstrated significant reductions in BF % (23.34 vs 25.21% in E1 and 25.29 vs 26.97% in E2) |  |
| **33** | - Stea et al, 2009[44] - NRCT - Strong | - 505 - 19 years - Conscripts | Norwegian army | - The intervention group was provided with information on the benefits of healthy eating habits, diet rich in vegetables, fruits and wholegrain bread, and low in fat content through 3 daily meals. The military chefs were trained to prepare and present healthy meals - In the control group, there was no change in the food regimen during military service (contained more fat, and fewer vegetables and fruits). | - 5 months - N/A - N/A - 5 months | - Energy intake, fruit and vegetables intake, whole grain intake, fibers intake. Significant increased total energy intake and reduced percentage of energy derived from total fat and proteins in the intervention group compared to control group after 5 months - The total intake of vegetables, fruits, berries and juice (increased (24 %) in the intervention group and (6%) in the control group). Wholegrain bread (increased to 29 %) and decreased 4.2%) in the intervention and control groups, respectively) and total dietary fiber significantly increased in comparison to the control (increased to 25.9 % and decreased 1.2% in the intervention and control groups, respectively) |  |
| **34** | - Dyrstad et al, 2006[43] - NRCT - Weak | - 107 - 19 years - Conscripts | Norwegian army | - One hour of strength training and 1 hour of endurance training in 4 different training sessions each week. Officers attended an educational course which gave practical and theoretical lessons in physical training - Standard care: received half the number of physical training sessions | - 10 weeks - 4 session per week (2 sessions for a control group) - Practical and theoretical sessions - 10 weeks & 10 months | - VO_2_ max, time to exhaustion (TTE), and the maximal numbers of push-ups, sit-ups, and chin-ups, 3-km running time and weight - VO_2_ max and TTE of the intervention group significantly increased (increased by 2.5% and 7.7%, respectively), with no changes in the control group. Overall significant increase in sit-ups (49 vs 29) and push-ups (32 vs 34) during basic training period with no significant differences between both groups. - No change in chin-ups and weight |  |
| **35** | - Maric et al, 2013[48] - Longitudinal study - Weak | - 120 - 19 years ± 6 months - Conscripts | Military academy in Serbia | - Physical education is taught by two regular physical education classes and 2 h on sports day - No control group | - 4 years - N/A - group classes - 1, 2, 3 and 4 years | - Pull-ups repetitions performed for 60 sec, sit-ups repetitions in 60 sec and 1,600-meter-run time (1,600) and obstacle course (OC) time - In year 2, there were significant improvements in the mean OC (131.2 ± 26.9 vs 139.7 ± 37.5) test and 1,600-meter run time (385.0 ± 37.8 vs 405.7±30.4) - In year 3, there was a significant decrease in OC test, SLJ and 1,600-meter run time as compared to the second year - In year 4, a slight increase in all three variables compared to year 3 (not significant). There was a significant increase in the percentage of cadets who do pull-ups over 14 reps compared to year 3 (29 vs 24) There was a significant increase in the percentage of cadets who do 50 sit-ups in year 4 compared to year 3 (89 vs 55) |  |
| **36** | - Hickey et al, 2012[46] - NRCT - Weak | - 35 - 21 years - Defense forces | Irish Defense Forces | - The intervention involved physical training, with 41 hours of physical education, front-loaded to the initial eight weeks of the course. - The control group was restricted to classroom-based and technical activity and recreational physical training in non-military settings. | - 12 weeks - N/A - N/A - 12 weeks (post intervention) | - 20-m shuttle-run distance, VO_2_ max, 2-mile run time, mean % HR max during the 10-km route march - 20-m shuttle-run distance time and VO_2_ max (49.8± 1 vs 52.4 ± 0.9), were significantly higher in both training and control groups compared to baseline. 2) 2-mile run time did not change significantly in both groups. 3) Mean % HR max during the 10-km route march was significantly higher in both groups after 12 weeks (71±1 and 83±1% vs 65±1 and 77± 1%). |  |
| **37** | - James et al, 1997[24] - Pretest-posttest study - Weak | - 32 - 32.05 years - Multiple military branches (Army, Navy, and Coast Guard) | US army | - The LE3AN Program consists of 3-week intensive inpatient weight loss program coupled with a 12-month outpatient follow-up. - Both generic and tailored - No control group | - 1 year - 3 weeks intensive treatment followed by weekly follow-up for 12 months - Lectures and exercise classes - Post intervention (3 weeks) and 6 months | - Weight, push-ups, sit ups and 2 miles run - The mean weight loss was 10 and 11 Ibs at post treatment and 26 and 29 Ibs at 6 month follow-up for men and women respectively in comparison to baseline - There were improvement in fitness measures such as push-ups, sit ups and 2 miles run |  |
| **38** | - Webber et al, 2012[34] - Retrospective study - Weak | - 276 - 29.0 ± 6.9 years - Air force | US Air Force base | - The intervention includes behavioral modification class (4-hour course) within 10 duty days targeting fitness, nutrition, and behavior change through a slide-based lecture and demonstration format. - the Be Well course is based on a core curriculum that is standardized across the USAF - No control group | - 10 days - 4-hour course within 10 duty days - Slide-based lecture and demonstration format - 10 weeks | - Total Fitness Assessment (FA) score, abdominal circumferences (AC), push-ups, sit-ups, Aerobic Fitness Time (seconds), and BMI between FA1 and FA2 - For males and females, the change in push-ups (42.3 ± 9.3 vs 37.5 ± 9.1 for males and 27.0 ± 7.6 vs18.6 ± 10.4 for females), sit-ups (48.3 ± 9.3 vs 42.3 ± 10.1 for males and 46.7 ± 7.6 vs 36.4 ± 11.7 for females), and aerobic fitness (746.4 ± 123.2 vs 795.5 ± 140.6 for males and 854.8 ± 71.6 vs 898.3 ± 111.3 for females), and total FA ( 79.3 ± 15.0 vs 69.6 ± 15.3 for males and 88.4 ± 6.8 vs 76.0 ± 14.4 for females) scores were significant - BMI significantly reduced for males only. |  |

*Note:* (N/A) means not stated, RCT: Randomised Control Trial, NRCT: Non Randomised Control Trial, BF%: Body Fat Percentage, WC: Waiste Circumference

*Age range is mentioned instead of mean age

The effect size was calculated if data needed are available
