# Supplementary material for: Dietary, physical activity, and weight management interventions among active-duty military personnel: a systematic review
Source: Mil Med Res. 2018 Dec 24;5:43. doi: 10.1186/s40779-018-0190-5 (PMC6309065; doi:10.1186/s40779-018-0190-5)
Supplement: Supplementary file 3 — Quality Assessment results for all included studies. (DOCX 22 kb) [file 40779_2018_190_MOESM3_ESM.docx]

Additional file 3

| **Author & year** | **Selection bias** | **Study design** | **Confounders** | **Blinding** | **Data collection methods** | **Withdrawals and drop-outs** | **Total rating** |
| --- | --- | --- | --- | --- | --- | --- | --- |
| Bingham et al, 2012 [52] | Weak | Strong | Strong | Moderate | Moderate | Weak | Weak |
| Dennis et al, 1999[14] | Strong | Strong | Strong | Moderate | Strong | Strong | Strong |
| Shrestha et al, 2013[35] | Weak | Strong | Strong | Moderate | Strong | Weak | Weak |
| Earles et al, 2007[29] | Moderate | Moderate | Strong | Moderate | Strong | Weak | Moderate |
| Hunter et al, 2007[30] | Moderate | Strong | Strong | Moderate | Strong | Strong | Strong |
| Smith et al, 2010 [33] | Weak | Strong | Weak | Moderate | Strong | Weak | Weak |
| James et al, 2001[26] | Weak | Strong | Strong | Moderate | Strong | Weak | Weak |
| Shay et al, 2009[32] | Weak | Strong | Strong | Moderate | Strong | Weak | Weak |
| Simpson et al, 2004[27] | Weak | Moderate | Strong | Moderate | Strong | Weak | Weak |
| Robbins et al, 2006[17] | Moderate | Strong | Strong | Moderate | Strong | Weak | Moderate |
| Mantzios et al, 2014[51] | Weak | Strong | Moderate | Strong | Strong | Moderate | Moderate |
| Bowles et al, 2006[28] | Moderate | Moderate | Weak | Moderate | Strong | Weak | Weak |
| Uglem et al, 2014[50] | Weak | Strong | Moderate | Moderate | Strong | Weak | Weak |
| Fiedler et al, 1999[25] | Weak | Strong | Weak | Moderate | Moderate | Weak | Weak |
| Maric et al, 2013[48] | Weak | Moderate | Weak | Moderate | Strong | Weak | Weak |
| Daniels et al, 1980[21] | Weak | Moderate | Weak | Moderate | Strong | Weak | Weak |
| McDoniel et al, 2008[31] | Weak | Strong | Moderate | Moderate | Strong | Strong | Moderate |
| Crombie et al, 2013[37] | Weak | Strong | Moderate | Moderate | Strong | Moderate | Moderate |
| Sammito et al, 2013[49] | Weak | Moderate | Weak | Moderate | Strong | Weak | Weak |
| Smith et al, 2012[36] | Weak | Strong | Moderate | Moderate | Strong | Weak | Weak |
| Trent et al, 1993[22] | Moderate | Moderate | Weak | Moderate | Strong | Moderate | Moderate |
| Stea et al, 2009[44] | Moderate | Strong | Moderate | Moderate | Moderate | Moderate | Moderate |
| Dyrstad et al, 2006[43] | Weak | Strong | Weak | Moderate | Strong | Weak | Weak |
| GAMBERA et al, 1999[23] | Weak | Strong | Strong | Moderate | Moderate | Weak | Weak |
| Thorsen et al, 2010[45] | Weak | Moderate | Weak | Moderate | Weak | Weak | Weak |
| Herzman-Harari et al, 2013[55] | Weak | Moderate | Weak | Moderate | Strong | Strong | Weak |
| Hofstetter et al, 2012[47] | Weak | Strong | Moderate | Moderate | Strong | Weak | Weak |
| Glick et al, 1975[56] | Weak | Moderate | Weak | Moderate | Strong | Weak | Weak |
| Hickey et al, 2012[46] | Weak | Strong | Weak | Moderate | Strong | Moderate | Weak |
| James et al, 1997[24] | Weak | Moderate | Weak | Moderate | Strong | Weak | Weak |
| Webber et al, 2012[34] | Weak | Moderate | Weak | Moderate | Strong | Weak | Weak |
| Buffington et al., 2016 [42] | Weak | Strong | Weak | Moderate | Strong | Strong | Weak |
| Sammito et al., 2016 [53] | Weak | Moderate | Weak | Moderate | Strong | Weak | Weak |
| Tomczak et al., 2016 [54] | Weak | Moderate | Weak | Moderate | Strong | Weak | Weak |
| Reppart et al., 1978 [40] | Weak | Moderate | Weak | Moderate | Strong | Weak | Weak |
| James et al., 1999 [39] | Weak | Moderate | Weak | Moderate | Strong | Strong | Weak |
| Davis 1996 [38] | Weak | Moderate | Weak | Moderate | Strong | Weak | Weak |
| Veverka et al., 2003 [41] | Moderate | Strong | Weak | Moderate | Strong | Strong | Moderate |

The quality assessment tool and dictionary is available at: https://www.nccmt.ca/knowledge-repositories/search/14
